# Synthesis and antibacterial activity of monocyclic 3-carboxamide tetramic acids

**DOI:** 10.3762/bjoc.9.224

**Published:** 2013-09-19

**Authors:** Yong-Chul Jeong, Mark G Moloney

**Affiliations:** 1 Chemistry Research Laboratory, University of Oxford, Mansfield Rd, University of Oxford, OX1 3TA, UK

**Keywords:** acylation, antibacterial, drug discovery, natural products, tetramate

## Abstract

A chemical library of carboxamide-substituted tetramates designed by analogy with antibacterial natural products, a method for their rapid construction, and the evaluation of their antibacterial activity is reported.

## Introduction

**Figure 1:**
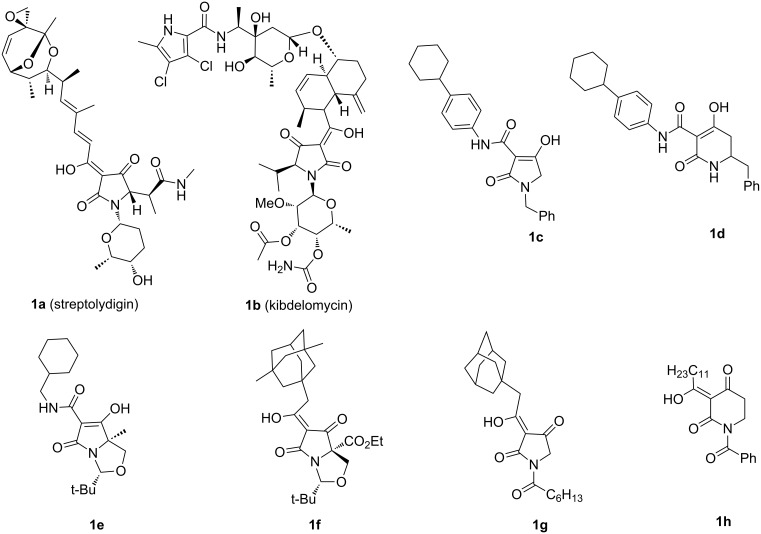
Some antibiotic natural and unnatural tetramic acids.

**Scheme 1:**
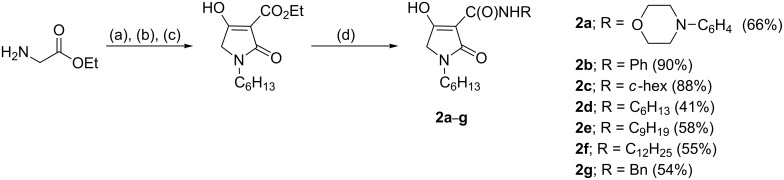
Synthesis of simple 3-carboxamide tetramic acids. Reaction conditions: (a) triethylamine (2.0 equiv), 1-bromohexane (0.5 equiv), EtOH, reflux; (b) monoethyl malonate (1.1 equiv), DCC (1.1 equiv), CH_2_Cl_2_, rt; (c) NaOMe (1.1 equiv), benzene, EtOH, reflux; (d) RNH_2_ (1.0 equiv), toluene, reflux.

**Scheme 2:**
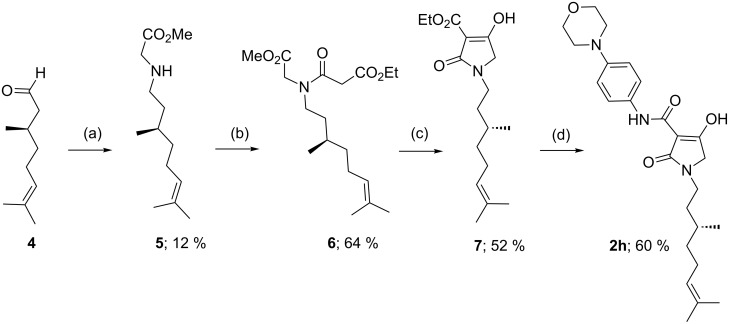
Synthesis of *N*-alkyl 3-carboxamide tetramic acid. Reaction conditions: (a) 1. glycine methyl ester∙HCl (1.0 equiv), Et_3_N (1.2 equiv), MgSO_4_ (2.0 equiv), THF, rt. 2. NaBH_4_ (2.0 equiv), MeOH, rt; (b) ethyl malonyl chloride (1.05 equiv), Et_3_N (1.2 equiv), CH_2_Cl_2_, rt; (c) KO*t*-Bu (1.1 equiv), THF, reflux; (d) amine (1.0 equiv), toluene, reflux; (e) butyl chloroformate (1.2 equiv), DMAP (2.2 equiv), CH_2_Cl_2_, rt.

**Scheme 3:**
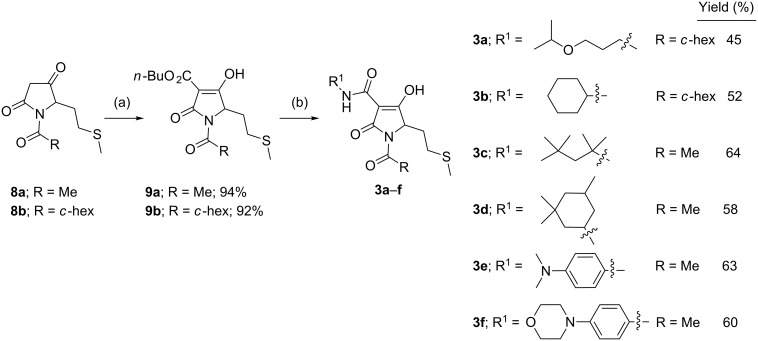
Synthesis of C(5)-alkyl 3-carboxamide tetramic acids. Reaction conditions: (a) butyl chloroformate (1.2 equiv), DMAP (2.2 equiv), CH_2_Cl_2_, rt; (b) RNH_2_ (1.0 equiv), toluene, reflux.

The discovery of new antibiotic families with novel modes of action is a promising way to overcome resistant or virulent bacteria, since novel modes of action might be expected to slow target-based endogenous resistance [1]. In this regard, natural products play a major role by providing a biologically validated starting point [2]. Recently discovered antibiotic lead compounds of major interest include platensimycin (a FabF inhibitor) [3], R207910 (an ATP synthase inhibitor) [4] and moiramide B (a bacterial acetyl-CoA carboxylase inhibitor) [5]. Both natural 3-acyltetramic acids, for example streptolydigin **1a** (bacterial RNA polymerase (RNAP) inhibitory activity) [6] and kibdelomycin **1b** (bacterial type II topoisomerase inhibitory activity) [7] and unnatural systems, such as 3-carboxamide tetramic acid **1c** and 3-carboxamide piperidine-2,4-dione **1d** (undecaprenyl pyrophosphate synthase (UPPS) inhibitory activity) [8] exhibit high levels of antibacterial activity (Figure 1). All these systems share a β-dicarbonyl core. A drug discovery programme inspired by these natural products, as promoted by Waldmann [9], was of interest to us. We have recently focused on the construction and evaluation of libraries derived from tetramic acid scaffolds and discovered that bicyclic 3-carboxamide **1e**, bicyclic 3-acyl **1f** and monocyclic 3-acyl **1g** exhibit a dual targeting ability at RNAP and UPPS, while 3-acyl piperidine-2,4-dione **1h** only targets UPPS [10]. Although tetramates are well-known as a core component in many natural products that continue to excite interest [11–14], we carried out a more detailed study of the synthesis, tautomeric behaviour and antibiotic activity of related monocyclic 3-carboxamide tetramic acid systems **2** and **3** (Schemes 1–3), the results of which are outlined below. In system **2**, the N(1) and C(5) substituents were chosen in order to probe the effect of the length of the *N*-alkyl chain on antibiotic activity. The substituents of system **3** were chosen in order to probe the effect of a C(3) substituent containing a sulfur heteroatom, which we had earlier seen results in enhanced antibacterial activity compared with the oxygen counterpart [10], for two types of amide substituent and a range of C(3) carboxamides.

## Results and Discussion

### Synthesis

The synthesis of the required tetramic acid systems **2a**–**g** (Scheme 1), **2h** (Scheme 2) and **3a**–**f** (Scheme 3) was achieved by Dieckmann cyclisation of the required *N*-alkyl-*N*-malonyl glycine (readily prepared from glycine). A similar strategy for the base-mediated cyclisation of *N*-acetoacetylamino acid esters leading to 3-acetyltetramates has been reported, which give N–H rather than *N*-alkyl systems [15–17]. Tetramate **7** was obtained from amino acid **6**, except that the key intermediate **5** was obtained by reductive amination (Scheme 2) of (*R*)-citronellal (**4**), which although proceeding in poor yield gave enough material with which to proceed [18]. By contrast, *N*-acyl derivatives could not be easily prepared by an equivalent approach because of the difficulty of a controlled double acylation on N(1). Although the synthesis of *N*-acetyl 3-alkoxycarbonyls from *N*-hydroxysuccinimide esters of *N*-acetylamino acids has been reported [19], we wished to exploit an alternative approach based upon C-acyloxylation of enolates followed by amine exchange, which had been shown to be very effective in a pyroglutamate series, since it offered synthetic simplicity and the potential for generality [20–21]. We found that an approach based upon direct acylation of methyl thioethers **8a**,**b** (these were readily obtained from the required *N*-acylmethionine by DCC/DMAP coupling with Meldrum’s acid and cyclisation under reflux) was possible, which made use of the high acidity of the tetramate system. Thus, conversion to the *n*-butyloxycarbonyl derivatives *N*-acyl **9a**,**b** by using 1.2 equivalents of butyl chloroformate along with 2.2 equivalents of 4-(dimethylamino)pyridine (DMAP) proceeded in good yields (over 90%) (Scheme 3) [10]. With the 3-alkoxycarbonyl tetramic acid core systems **7** and **9** in hand, conversion to 3-carboxamides **2a**–**g** and **3a**–**f** by direct ester–amide exchange under reflux in toluene was readily achieved, providing access to a range of amides in good to excellent yield (Schemes 1–3). This process neatly complements a strategy we had earlier used for the introduction of amine substituents in pyroglutamates by a conjugate addition of amines [22].

### Tautomerism

**Figure 2:**
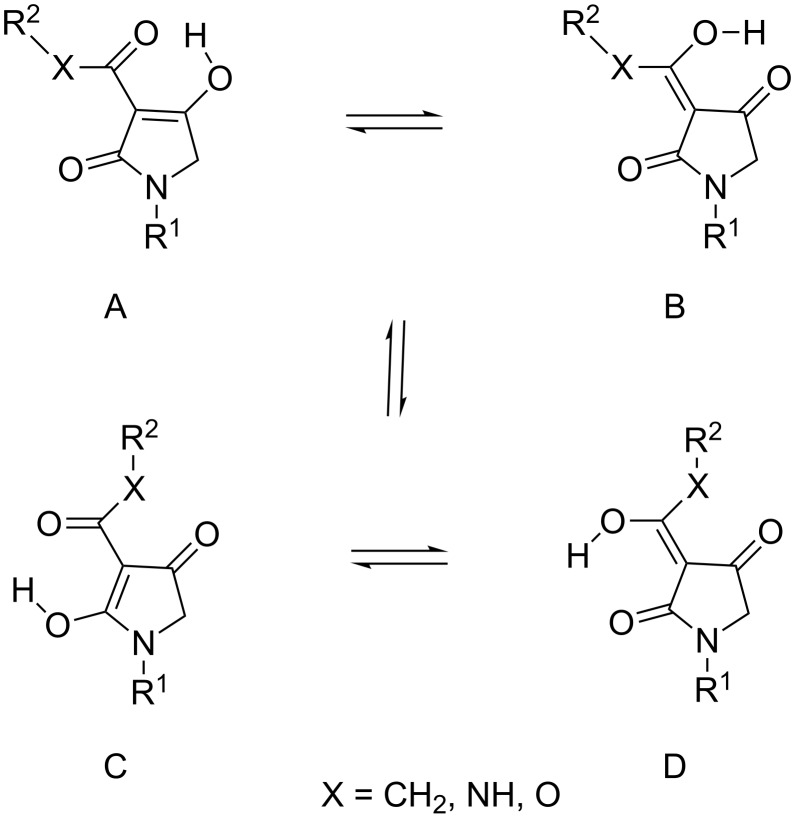
Tautomerism of tetramates.

Tautomerism in tricarbonyl 3-acyltetramate systems is known to be complex and strongly dependent on the identity of the side chain acyl group [23]. 3-Acyl (X = CH_2_) [10,24–25], 3-carboxamide (X = NH) [8,10] and 3-alkoxycarbonyl (X = O) tetramic acids (Figure 2) have been found to exist as a pair of external conformers (AB and CD) in slow equilibrium (AB
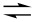
CD), each consisting of a pair of internal tautomers in rapid equilibrium (A
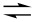
B and C
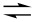
D). The tautomerisation of 3-acyltetramic acids has been shown to be mainly affected by substitution on N(1) rather than the functionalities on the 3-acyl and C(5) positions. Thus, the dominant tautomer of N-unsubstituted and *N*-alkyltetramates is D, while *N*-acyltetramates exist as a mixture of external tautomers AB and D in approximately equal ratio [23]. By contrast, it was found that the tautomerisation of 3-carboxamides and 3-alkoxycarbonyls was not affected by substitution on N(1). Therefore, the dominant tautomer of 3-carboxamide tetramates is tautomer A (over 80%) with a minor contribution of tautomer D, while 3-alkoxycarbonyltetramates exist as only tautomer A (>99%). In order to understand this phenomenon, the ground state energy of simplified 3-carboxamides **11a**,**b** and 3-alkoxycarbonyls **12a**,**b** was calculated and compared with that of 3-acyl derivatives **10a**,**b** (Table 1). To the best of our knowledge, such a detailed analysis has not been previously reported. In the calculation for *N*-alkyl 3-acyltetramate **10a**, the ground-state energies of tautomers B and D, which shows preference for tautomer D, is considerably lower than those of tautomers A and C. This outcome supports experimental NMR observations (>80% of tautomer D and <20% of tautomer B, Table 1, entry 1). On the other hand, the ground state energy of tautomer C of *N*-acyl **10b** is considerably higher than that of tautomers A, B and D, also supporting the NMR observations (tautomer AB:D = about 50:50, Table 1, entry 2). However, tautomer A of 3-carboxamides was the most stable for both of *N*-alkyl tetramate **11a** and *N*-acyl tetramate **11b** (>80% of tautomer A and <20% of tautomer D, Table 1, entry 3 and entry 4), while for 3-alkoxycarbonyltetramates **12a**,**b**, tautomer A was the most stable, and significantly more stable than tautomers B–D. These finding support the conclusion that 3-alkoxycarbonyls exist only as tautomer A (Table 1, entry 5 and entry 6).

**Table 1:** Calculated energy of the ground state of 3-acyltetramic acids **10a**,**b**, 3-carboxamide tetramic acids **11a**,**b** and 3-alkoxycarbonyl tetramic acids **12a**,**b**.

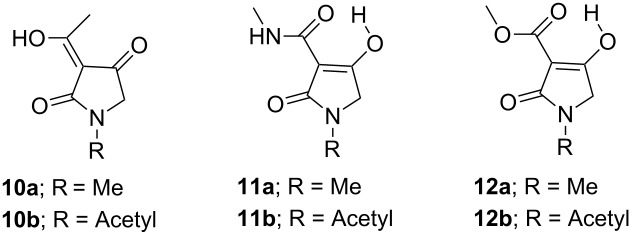					
Entry	Compound	Calcd relative energy (kcal/mol)^a,b^			

		Form A	Form B	Form C	Form D

1	**10a**	+4.16	+1.61	+4.30	0
2	**10b**^c^	+1.59	+0.17	+5.62	0
3	**11a**	−0.51	+0.42	+1.74	0
4	**11b**	−0.91	−0.15	+3.82	0
5	**12a**	−3.58	+1.75	−1.41	0
6	**12b**	−6.24	−0.64	+3.20	0

^a^The energy difference between each tautomer related to tautomer D. ^b^Calculated by using DFT B3LYP (6-31G*) in Spartan 02. ^c^Reported in our previous paper [10].

### Antibacterial activity

The antibiotic activities of tetramates **2a**–**h** and **3a**–**f** along with analogues **1c** and **1d** (reported by Novartis [8]) were determined against 4 species of Gram-positive bacteria, consisting of 4 strains of *Staphylococcus aureus*, including a methicillin-resistant strain (MRSA, S2), vancomycin susceptible *Enterococcus faecalis* (VSE, E1), vancomycin resistant *E. faecium* (VanA VRE, E2)), and 2 strains of *Streptococcus pneumoniae*, including multi-drug resistant strain (MDRSP, P9), as well as 3 species of Gram-negative bacteria, consisting of *Pseudomonas aeruginosa* and 2 strains of *Haemophilus influenzae*, including an efflux-negative strain, and 2 strains of *Escherichia coli*, including an efflux-negative strain (Table 2). Relevant physicochemical properties of the analogues are also shown in Table 3. These were used to elaborate structure–activity relationships (SAR) [26]. In the assay against Gram-negative bacteria, no activity against *E. coli* and *P. aeruginosa* was found (MIC > 64 µg/mL, data not shown), consistent with the inactivity of 3-acyl and 3-carboxamide tetramic acids against these strains seen earlier [7–8,10]. This result is most likely explained by their poor cell permeability as a result of their hydrophobic character [14,27]. However, activities against another Gram-negative bacterium, *H. influenzae*, were found, the magnitude of which depended on the substituent. An SAR consistent with transportation by the efflux pump was found [28]. Analogues **2e**,**h** and **1c**,**d**, for which activity differences between efflux-positive (H3) and negative (H4) *H. influenza* strains are large, are more lipophilic compared with other active analogues (rel-PSA < 13.5, c log P > 2.79 and c log D (7.4) > 1.41). On the other hand, analogues **2** and **3** show a broad-spectrum activity against Gram-positive bacteria, although the activity depends on the substituent identities. Importantly, the variation of antibacterial activity for analogues **2** and **3** was less pronounced in the resistant strains MRSA, VRE and MDRSP (normally less than 8 times variation, with the exception of analogues **2e** and **3b**), while the activities of Novartis analogue **1c** against MRSA [8], amoxicillin against MDRSP and ciprofloxacin against MRSA and VRE were significantly lower (more than 250 times compared with the activity against the most sensitive strain). Among the various substituent groups, *N*-alkyl phenyl derivatives **2a**,**b**,**h** were active, while *N*-acetyl phenyl derivatives **3e**,**f** were inactive or only very weakly active. This SAR might be accounted for by their physicochemical properties: the less lipophilic character of *N*-acetyl **3e**,**f** (rel-PSA > 17.0%, c log P < 0.80 and c log D (7.4) < −1.20) compared with those of *N*-alkyl **2a**,**b**,**h** (rel-PSA < 15.0%, c log P > 1.77 and c log D (7.4) > 0.41) might make penetration of the bacterial cell membrane more difficult. However, 3-carboxamides **2c**–**g** and **3a**–**d**, all possessing alkyl substituents, including a benzyl group on the amide function, are also active to Gram-positive strains. Furthermore, the activities of *n*-alkyl **2d**–**f** depended on the chain length, with a marked drop-off in activity for the longer chain **2f**. In addition, the activity of the more lipophilic analogues **2a**,**d**–**f**,**h** (PSA ≤ 13.7%, c log P ≥ 1.69 and c log D (7.4) ≥ 0.23) in the presence of 2.5% horse blood was shifted to high MICs even though that of less lipophilic analogues **2c**,**g** and **3a**–**e** (PSA ≥ 13.7%, c log P ≤ 1.84 and c log D (7.4) ≤ −0.04) was maintained. This serum-protein binding significantly affected the activity against *S. aureus* S26, since almost all analogues showed inactivity (MIC > 64 µg/mL) in the presence of 10% human serum with the exception of **2a** (32 µg/mL) and **2b** (64 µg/mL) (data not shown).

**Table 2:** In vitro antibiotic activity (MIC, µg/mL) of tetramic acids.^a,b^

	S1	S26	S4	S2	E1	E2	P1	P9	P9B	H3	H4

**2a**^c^	4	8	8	8	8	8	16	8	32	16	4
**2b**^c^	1	1	1	2	2	4	4	4	−^d^	2	0.5
**2c**^c^	8	8	8	8	8	8	16	8	8	4	0.25
**2d**^c^	2	2	2	2	2	2	4	1	4	1	0.12
**2e**^c^	2	16	16	16	8	0.5	4	1	4	>64	≤0.1
**2f**	>64	>64	>64	>64	>64	>64	16	8	16	>64	>64
**2g**	8	8	8	8	16	8	64	16	8	4	0.25
**2h**	2	2	2	2	2	2	8	4	>64	>64	8

**3a**	>64	>64	32	>64	16	16	8	8	8	>64	>64
**3b**	4	8	2	8	1	2	0.5	0.5	0.5	8	1
**3c**	8	32	32	16	8	16	8	8	8	32	4
**3d**	8	64	32	32	8	16	8	8	4	64	4
**3e**	>64	>64	>64	>64	>64	>64	64	64	64	>64	64
**3f**	>64	>64	>64	>64	>64	>64	>64	>64	>64	>64	>64

**1c**^c^	0.1	0.12	1	**64**	≤0.1	≤0.1	2	2	2	>64	0.5
**1d**	8	64	32	64	32	4	64	64	>64	>64	16
**Line**^c^	2	2	2	2	2	2	1	0.5	0.5	16	4
**Cip**	0.1	0.5	0.12	**16**	1	**32**	1	1	1	0.5	≤0.1
**Amox**	−^d^	−^d^	−^d^	−^d^	−^d^	−^d^	>0.03	**8**	−^d^	−^d^	−^d^
**Caz**	8	16	16	−^d^	−^d^	−^d^	−^d^	−^d^	−^d^	−^d^	−^d^

^a^Abbreviation; **S1**; *S. aureus* 1, ATCC13709 in vivo (methicillin sensitive), **S26**; *S. aureus* 26, ATCC25923 (vancomycin susceptible), **S4**; *S. aureus* 4, Oxford, **S2**; *S. aureus* 2, (MRSA in vivo), **E1**; *E. faecalis* 1, ATCC29212 VanS (vancomycin susceptible), **E2**; *E. faecium* 1, VanA (vancomycin resistant), **P1**; *S. pneumonia* 1, ATCC49619 (erythromycin susceptible), **P9**; *S. pneumonia* 9, (multi-drug resistant), **P9B**; *S. pneumonia* 9 in presence of 2.5% horse blood, **H3**; *H. influenzae* 3, ATCC31517 MMSA, **H4**; *H. influenzae* 4, LS2 Efflux knock out, **Line**; linezolid**, Cip**; ciprofloxacin, **Amox**; amoxicillin, **Caz**; ceftazidime. ^b^All analogues are inactive (MIC > 64 µg/mL) against *E. coli* 1, ATCC25922 (non pathogenic strain), *E. coli* 50, Ec49 No efflux and *P. aeruginosa* 1, ATCC27853. ^c^The activity was reported in our previous publication [10]. ^d^Not determined.

**Table 3:** Physicochemical properties of 3-carboxamide tetramic acids.^a,b^

	MW	MSA	PSA	rel-PSA	c log P	c log D (7.4)	HD/HA	RB

**2a**^c^	387	599	82.1	13.7	1.77	0.41	2/5	8
**2b**^c^	302	462	69.6	15.1	1.85	0.64	2/3	7
**2c**^c^	308	509	69.6	13.7	1.28	−0.15	2/3	7
**2d**^c^	310	540	69.6	12.9	1.69	0.23	2/3	11
**2e**^c^	353	632	69.6	11.0	2.88	1.42	2/3	14
**2f**	395	725	69.6	9.6	4.07	2.61	2/3	17
**2g**	316	492	69.6	14.1	1.47	−0.04	2/3	8
**2h**	442	691	82.1	11.9	2.79	1.41	2/5	9

**3a**	427	662	95.9	14.5	0.90	−1.32	2/5	10
**3b**	409	613	86.7	14.1	1.84	−0.19	2/4	6
**3c**	371	590	86.7	14.7	0.84	−1.13	2/4	7
**3d**	383	586	86.7	14.8	1.06	−0.96	2/4	5
**3e**	377	530	90.0	17.0	0.80	−1.20	2/5	6
**3f**	419	577	99.2	17.2	0.46	−1.54	2/6	6

**1c**	390	568	69.6	12.3	3.51	2.03	2/3	5
**1d**	405	591	78.4	13.3	3.63	2.96	3/3	5

^a^MW; molecular weight, MSA; molecular surface area, PSA; polar surface area, %PAS; relative polar surface area = (PSA/MSA) × 100, c log P; calculated partition coefficient, c log D (7.4); calculated distribution coefficient at pH 7.4, HD; hydrogen-bond donor count, HA; hydrogen-bond acceptor count, RB; rotatable bond count. ^b^Tautomer A was selected for the calculation. ^c^Reported in our previous publication [10].

After screening to find the most active compound, tetramate **2b** was selected for a detailed investigation and its antibiotic activity against various drug-resistant strains was further evaluated with reference antibiotics (Table 4). Tetramate **2b** was found to be active against virulent and resistant strains, such as methicillin and fluoroquinolone-resistant *S. aureus* and penicillin and/or erythromycin-resistant *S. pneumoniae*. Remarkably, tetramate **2b** maintained the activities against all the strains and was highly effective against erythromycin resistant *S. pneumoniae* Pn31 (MIC = 0.125 µg/mL). By way of comparison, the activities of moxifloxacin (a fourth generation fluoroquinolone) to *S. aureus* Sa18 (MIC = 32 µg/mL), amoxicillin (β-lactam) to *S. pneumoniae* Pn7, Pn10 and Pn11 (MIC = 4 µg/mL), erythromycin to *S. pneumoniae* Pn7, Pn10, Pn19, Pn21 and Pn31 (MIC ≥ 4 µg/mL) and ceftazidime (a third generation cephalosporin) to *S. aureus* Sa5, Sa18 and Sa40 (MIC ≥ 16 µg/mL) were substantially decreased.

**Table 4:** Antibiotic activity of tetramic acid **2b**.^a^

Strains	Phenotype	MIC (µg/mL)					

		**2b**	Moxi	Amox	Ery	Vanco	Caz

*S. pneumoniae* Pn7	EryR	2	0.125	**4**	**16**	0.25	−^c^
*S. pneumoniae* Pn10	PenR, EryR	2	0.125	**4**	**>32**	0.5	−^c^
*S. pneumoniae* Pn11	PenR	2	0.125	**4**	<0.03	0.25	−^c^
*S. pneumoniae* Pn19	EryR	2	0.06	0.06	**>32**	0.5	−^c^
*S. pneumoniae* Pn21	EryR	2	0.06	0.125	**4**	0.25	−^c^
*S. pneumoniae* Pn31	EryR	0.13	<0.03	<0.03	**16**	0.5	−^c^

*S. aureus* Sa5	ermR PK2^b^	1	≤0.03	−^c^	−^c^	−^c^	**32**
*S. aureus* Sa18	FQR	1	**32**	−^c^	−^c^	−^c^	**16**
*S. aureus* Sa40	mecA^b^	1	≤0.03	−^c^	−^c^	−^c^	**32**

^a^Abbreviation: EryR; erythromycin resistant, PenR; penicillin resistant, FQR; fluoroquinolone resistant, Moxi; moxifloxacin, Amox; amoxicillin, Ery; erythromycin, Caz; ceftazidime. ^b^Methicillin-resistant strain. ^c^Not determined.

Lipophilic efficiency (LipE) has been used to assess the suitability of drug candidates as CB agonists [29], and the usage of a similar calculation for the data presented in this work (with LipE = pMIC(nM) − c Log P) (see Table S1 and Figure S1 in Supporting Information File 1; pMIC values were calculated according to a literature protocol [30]), facilitated the identification of compounds with potential for optimisation. According to Figure S1, strongly active compounds can be found at c log P values of 1–2, 3 or 4, and for the highly susceptible strain H4, for example, compounds of interest would be **2b**–**e** and **2g**.

## Conclusion

We have prepared monocyclic 3-carboxamide tetramic acids from 3-alkoxycarbonyl tetramic acids based on a direct ester–amide exchange by using butyl chloroformate with DMAP, thereby providing a general access to this type of system. The tautomerization of 3-alkoxycarbonyl and 3-carboxamide tetramic acids compared to 3-acyltetramic acids has been investigated. It has been found that 3-alkoxycarbonyl and 3-carboxamide tetramic acids prefer tautomer A, while the preference of 3-acyltetramic acids depends on the N(1)-functionality. Of particular interest is that 3-carboxamide analogues, especially **2b**, have shown bioactivity against various Gram-positive bacteria including clinically resistant strains such as MRSA, fluoroquinolone-resistant *S. aureus*, MDRSP, penicillin and erythromycin-resistant *S. pneumonia* and VRE as well as Gram-negative *H. influenzae*. Further optimisation, especially for overcoming high plasma-protein binding, is warranted but these compounds may be suitable for an evaluation for topical use [31–32]. Significantly, these results suggest that a drug discovery approach based upon deconstruction–reconstruction inspired by suitable natural products with demonstrable biological activity provides a route for the rapid assembly of compound libraries, which, even if not fully optimized, provide a useful starting point for further elaboration.

## Experimental

**General.** Melting points were determined with a Stuart Scientific SMP1 melting point device and are uncorrected. The ^1^H and ^13^C NMR spectra were obtained by using a Bruker Avance AV400 (400 MHz and 100 MHz, respectively) with residual solvent peaks as the internal reference. Mass spectra (MS) and high-resolution mass spectra (HRMS) were obtained with Micro Mass LCT and GCT spectrometers under the conditions of electrospray ionization (ESI) and chemical ionization (CI) respectively, and values were reported as a ratio of mass to charge in Daltons.

**Synthesis.** The synthesis of monocyclic precursor tetramate compounds from glycine has been reported in our previous publication [33].

**Calculations.** Density Functional B3LYP (6-31G*) in Spartan 02 was used for the calculation of the energy in equilibrium geometry at ground state. MarvinSketch Version 5.3.8. (http://www.chemaxon.org) was used for the calculation of the van der Waals molecular surface area (MSA), the polar surface area (PSA), the calculated partition coefficient (c log P) under VG method, the calculated distribution coefficient at pH 7.4 [c log D (7.4)] under VG method, the hydrogen-bond donor count, the hydrogen-bond acceptor count, and the rotatable bond count.

**Antibacterial activity.** Antibiotic activity was measured by using standard methodology (Clinical and Laboratory Standards Institute. Methods for antimicrobial susceptibility test for bacteria that grow aerobically, approved standard M7-A7, 7th ed., CLSI, Wayne, PA, 2006): compounds were diluted in DMSO to obtain 2.56 mg/mL, then 100 µL were diluted in Mueller-Hinton broth to 0.256 mg/mL and assayed against the bacterial panel by incubation in 96-well microplates at 37 °C for 24 h. The MIC was determined by visually reading the first concentration where no growth (no turbidity) appeared.

## Supporting Information

File 1 Experimental details, calculated energies (Spartan) for selected compounds and NMR spectra.
