# Supplementary material for: Synthesis and antibacterial activity of monocyclic 3-carboxamide tetramic acids
Source: Beilstein J Org Chem. 2013 Sep 19;9:1899–906. doi: 10.3762/bjoc.9.224 (PMC3817595; doi:10.3762/bjoc.9.224)
Supplement: File 1 — Experimental details, calculated energies (Spartan) for selected compounds and NMR spectra. [file Beilstein_J_Org_Chem-09-1899-s001.pdf]

Supporting Information

for

Synthesis and antibacterial activity of monocyclic 3-carboxamide tetramic acids

Yong-Chul Jeong and Mark G. Moloney*

Address: Chemistry Research Laboratory, University of Oxford, Mansfield Rd, University of Oxford, OX1 3TA, UK

E-mail: Mark G. Moloney - mark.moloney@chem.ox.ac.uk

*Corresponding author

Experimental details, calculated energies (Spartan) for selected compounds and NMR spectra

Synthesis of compound **5**

To a solution of glycinemethyl ester hydrochloride (4.0 g, 32.4 mmol) in THF (150 mL) were added $MgSO_4$ (7.8 g, 64.8 mmol), (*R*)-citronellal (**4**, 5.0 g, 32.4 mmol) and triethylamine (3.9 g, 38.9 mmol). The reaction was stirred at room temperature overnight. Then the reaction mixture was filtered and the solvent was evaporated to give the crude imine. The crude imine was dissolved in methanol (70 mL), and sodium borohydride (2.9 g, 77.8 mmol) was slowly added to the solution at 0 °C. The reaction was stirred at room temperature for 1 hour, and then quenched with 1 N NaOH (150 mL). The mixture was extracted with ethyl acetate (100 mL x 3 times). The organic layer was dried over $MgSO_4$ and concentrated in vacuo. The residue was purified by flash column chromatography to afford pure compound **5** (880 mg, 3.87 mmol, 12% yield) as an oil. ^{1}H NMR (400 MHz, $CDCl_3$) 5.08 (t, 1H, *J* = 7.2 Hz, $C\underline{H}{=}C$), 3.72 (s, 3H, $OC\underline{H}_3$), 3.40 (s, 2H, $COC\underline{H}_2NH$), 2.65-2.54 (m, 2H, $NHC\underline{H}_2CH_2$), 2.03-2.88 (m, 2H, $CH_2C\underline{H}_2CH$), 1.67 (s, 3H, $C(C\underline{H}_3)_2$), 1.59 (s, 3H, $C(C\underline{H}_3)_2$), 1.56-1.43 (m, 2H, $CH(CH_3)C\underline{H}_2(CH_2)_2CH$), 1.36-1.25 (m, 2H, $NHCH_2C\underline{H}_2$), 1.19-1.10 (m, 1H, $C\underline{H}(CH_3)$), 0.88 (d, 3H, *J* = 6.8 Hz, $CH(C\underline{H}_3)$); ^{13}C NMR (100 MHz, $CDCl_3$); 172.98 ($\underline{C}{=}O$), 131.12 ($CH{=}\underline{C}(Me)_2$), 124.71 ($\underline{C}H{=}C(Me)_2$), 51.66 ($O\underline{C}H_3$), 50.85 ($CO\underline{C}H_2NH$), 47.52 ($NH\underline{C}H_2CH_2$), 37.14 ($\underline{C}H_2CH(Me)\underline{C}H_2$), 30.41 ($\underline{C}H(Me)$), 25.65 ($C(\underline{C}H_3)_2$), 25.41 ($CH\underline{C}H_2$), 19.47 ($CH(\underline{C}H_3)$), 17.57 ($C(\underline{C}H_3)_2$); MS ($ES^+$) 228.19 (M + H), 250.20 (M + Na).

Synthesis of compound **6**

To a mixture of compound **5** (880 mg, 3.87 mmol) and triethylamine (500 mg, 4.95 mmol) in CH_2Cl_2 (50 mL) was added ethyl malonyl chloride (640 mg, 4.26 mmol) and stirred at room temperature for 1 hour. The mixture was filtered, concentrated in vacuo, and flash column chromatography gave compound **6** (840 mg, 64% yield) as an oil. ^{1}H NMR (400 MHz, $CDCl_3$, mixture of rotamers, a; major, b; minor) 5.07 (t, 1H, J = 6.8 Hz, $C\underline{H}$), 4.24-4.16 (m, 2H, $OC\underline{H}_2CH_3$), 4.08 (s, 2H, $NC\underline{H}_2CO$ a), 4.06 (s, 2H, $NC\underline{H}_2CO$ b), 3.78 (s, 3H, $OC\underline{H}_3$ b), 3.73 (s, 3H, $OC\underline{H}_3$ a), 3.49 (s, 2H, $COC\underline{H}_2CO$ a), 3.38 (s, 2H, $COC\underline{H}_2CO$ b), 3.44-3.26 (m, 2H $NC\underline{H}_2CH_2$), 2.04-1.89 (m, 2H, $CHC\underline{H}_2$), 1.68 (s, 3H, $C(C\underline{H}_3)_2$), 1.60 (s, 3H, $C(C\underline{H}_3)_2$), 1.56-1.13 (m, 7H, $C\underline{H}_2CH(CH_3)C\underline{H}_2$ and $OCH_2C\underline{H}_3$), 0.92-0.89 (m, 3H, $CH(C\underline{H}_3)$); ^{13}C NMR (100 MHz, $CDCl_3$, mixture of rotamers, a; major, b; minor) 169.53 (ester $\underline{C}$=O b), 169.39 (ester $\underline{C}$=O a), 167.37 (ester $\underline{C}$=O b), 167.24 (ester $\underline{C}$=O a), 166.34 (amide $\underline{C}$=O b), 166.03 (amide $\underline{C}$=O a), 131.65 ($CH=\underline{C}(Me)_2$), 124.58 ($\underline{C}H=C(Me)_2$ b), 124.14 ($\underline{C}H=C(Me)_2$ a), 61.48 ($O\underline{C}H_2CH_3$), 52.53 ($O\underline{C}H_3$ b), 52.13 ($O\underline{C}H_3$ a), 49.85 ($N\underline{C}H_2C=O$), 48.01 ($\underline{C}H_2$), 47.39 ($\underline{C}H_2$), 45.88 ($\underline{C}H_2$), 41.49 ($\underline{C}H_2$), 40.82 ($\underline{C}H_2$), 36.89 ($\underline{C}H_2$), 36.76 ($\underline{C}H_2$), 35.40 ($\underline{C}H_2$), 33.95 ($\underline{C}H_2$), 30.34 ($\underline{C}H(CH_3)$ b), 30.24 ($\underline{C}H(CH_3)$ a), 25.67 ($C(\underline{C}H_3)_2$), 25.31 ($CH_2\underline{C}H_2CH$), 19.34 ($CH(\underline{C}H_3)$), 17.61 ($C(\underline{C}H_3)_2$), 14.07 ($OCH_2\underline{C}H_3$); MS (ES^+) 342.25 (M + H), 364.19 (M + Na).

Synthesis of compound **7**

To the solution of compound **6** (830 mg, 2.53 mmol) in THF (20 ml) was added potassium *tert*-butoxide (310 mg, 2.79 mmol) and the mixture wsa heated under reflux for 3 hours. After the completion of the reaction, the mixture was cooled to room temperature and 1 N HCl (50 mL) was added. The mixture was extracted with

dichloromethane (50 mL x 3 times). The organic layer was dried over $MgSO_4$ and concentrated in vacuo. The residue was purified by flash column chromatography to afford pure compound **7** (405 mg, 1.31 mmol, 52% yield) as a solid (mp 91 ^oC). ^1H NMR (400 MHz, $CDCl_3$) 5.06 (t, 1H, *J* = 6.4 Hz, $C\underline{H}=C$), 4.37 (q, 2H, *J* = 6.8 Hz, $OC\underline{H}_2CH_3$), 3.94 (s, 2H, C5), 3.52-3.33 (m, 2H, $NC\underline{H}_2$), 2.00-1.88 (m, 2H, $CHC\underline{H}_2$), 1.66 (s, 3H, $C(C\underline{H}_3)_2$), 1.59 (s, 3H, $C(C\underline{H}_3)_2$), 1.56-1.14 (m, 8H, $C\underline{H}_2C\underline{H}(CH_3)C\underline{H}_2$ and $OCH_2C\underline{H}_3$), 0.93 (d, 3H, *J* = 6.0 Hz, $CH(C\underline{H}_3)$); ^{13}C NMR (100 MHz, $CDCl_3$); 183.42 (C4), 167.42 (C2), 165.85 (ester $\underline{C}=O$), 131.33 ($CH=\underline{C}(Me)_2$), 124.49 ($\underline{C}H=C(Me)_2$), 99.83 (C3), 61.28 ($O\underline{C}H_2CH_3$), 48.87 (C5), 39.43 ($N\underline{C}H_2$), 36.89 ($CH(Me)\underline{C}H_2CH_2CH$), 35.08 ($NCH_2\underline{C}H_2$), 29.95 ($\underline{C}H(Me)$), 25.63 ($C(\underline{C}H_3)_2$), 25.27 ($C=CH\underline{C}H_2$), 19.29 ($CH(\underline{C}H_3)$), 17.60 ($C(\underline{C}H_3)_2$), 14.13 ($OCH_2\underline{C}H_3$); MS ($ES^-$); 308.21 (M − H); HRMS (M − H) calcd for $C_{17}H_{26}N_1O_4$, 308.1867; found, 308.1866.

Synthesis of 1-acetyl-5-(2-(methylthio)ethyl)pyrrolidine-2,4-dione (**8a**)

To a solution of *N*-acetyl-L-methionine (5.00 g, 26.1 mmol) in dichloromethane (150 mL) was added Meldrum's acid (1.1 equiv), DMAP (1.2 equiv) and DCC (1.2 equiv), and stirred overnight at room temperature. The mixture was filtered and the solution was washed with 5% aqueous $KHSO_4$ and saturated NaCl. The organic layer was evaporated to give a solid mass. The residue was dissolved in acetone and filtered for removing impurity and the solution was evaporated. The intermediate was obtained as a solid from precipitation in ethyl acetate and petrol solution. Then the solid was dissolved in ethyl acetate, and heated under reflux for 2 hours. After completion of the reaction, the solution was evaporated to give compound **6a** (5.0 g, 26.1 mmol, 31% yield) as a white solid (mp 95 °C). ^1H NMR (500 MHz, $CDCl_3$, enol: keto = 35: 65) 5.06 (s, 1H, C3 enol), 4.69 (dd, 1H, J_1 = 6.0 Hz, J_2 = 3.0 Hz, C5 enol),

4.50 (d, 1H, *J* = 7.0 Hz, C5 keto), 3.52 (dd, 1H, J_1 = 22.5 Hz, J_2 = 2.0 Hz, C3 keto), 3.25 (d, 1H, *J* = 22 Hz, C3 keto), 2.60 (s, 3H, C7 keto), 2.55-2.20 (m, 4H, C8 and C9), 2.51 (s, 3H, C7 enol), 2.07 (s, 3H, C10 enol), 1.91 (s, 3H, C10 keto); ^{13}C NMR (125 MHz, $CDCl_3$) 202.5 (C4 keto), 178.5 (C4 enol), 172.1 (C2 enol), 170.8 (C2 keto), 170.4 (C6 enol), 170.0 (C6 keto), 94.9 (C3 enol), 65.5 (C5 enol), 59.1 (C5 keto), 43.2 (C3 keto), 28.8 (C9 keto), 28.4 (C9 enol), 28.3 (C8 enol), 27.3 (C8 keto), 26.1 (C7 enol), 25.1 (C7 keto), 15.3 (C10 enol), 14.5 (C10 keto); MS (ES^-) 214.04; HRMS (M − H) calcd for $C_9H_{12}N_1O_3S_1$; 214.0543; found; 214.0547.

Synthesis of 1-(cyclohexanecarbonyl)-5-(2-(methylthio)ethyl)pyrrolidine-2,4-dione (**8b**)

Compound **8b** was prepared by using the same method as for compound **3a**. Yield; 49%; mp 121 °C; ^{1}H NMR (400 MHz, $CDCl_3$, enol: keto form = 20: 80) 5.03 (s, 1H, C3 enol), 4.67 (dd, 1H, J_1 = 6.0 Hz, J_2 = 2.8 Hz, C5 enol), 4.52 (d, 1H, *J* = 6.8 Hz, C5 keto), 3.55-3.48 (m, 2H keto and 1H enol, C3 and C10 keto, and C10 enol), 3.25 (d, 1H, *J* = 22 Hz, C3 keto), 2.53-2.15 (m, 4H, C6 and C7), 2.08 (s, 3H, C8 enol), 1.90 (s, 3H, C8 keto), 2.01-1.09 (m, 10H, C11-C15); ^{1}H NMR (400 MHz, MeOD, only enol form) 4.88 (brs, 2H, C3 and OH), 4.69-4.677 (m, 1H, C5), 3.59-3.51 (m, 1H, C10), 2.38-2.08 (m, 4H, C6 and C7), 2.04 (s, 3H, C8), 1.94-1.20 (m, 10H, C11-C15); ^{13}C NMR (100 MHz, MeOD, only enol form); 180.1 (C4), 177.9 (amide carbonyl), 173.3 (amide carbonyl), 66.3 (C5), 45.1 (C10), 31.4 (CH_2), 29.7 (CH_2), 29.5 (CH_2), 28.6 (CH_2), 27.2 (CH_2), 27.2 (CH_2), 26.8 (CH_2), 15.5 (C8); MS (ES^-) 282.11 (M − H); HRMS (M − H) calcd for $C_{14}H_{20}N_1O_3S_1$, 282.1169; found, 282.1171.

Synthesis of **9a**

To a solution of tetramic acid **8a** (3.0 g, 13.9 mmol) and DMAP (3.7 g, 30.7 mmol) in dichloromethane (100 mL) was slowly added butyl chloroformate (250 mg, 1.78 mmol) under nitrogen atmosphere at 0 °C, and the mixture stirred overnight at room temperature under nitrogen atmosphere. After completion of the reaction, the mixture was washed with 2 M HCl (50 mL x 2 times). The organic layer was dried over $MgSO_4$ and evaporated in vacuo. The residue was purified by flash column chromatography to give tetramic acid **9a** (4.11 g, 13.1 mmol, 94% yield) as an oil. ^{1}H NMR (400 MHz, $CDCl_3$) 4.81 (t, 1H, *J* = 4.0 Hz, C5), 4.28 (t, 2H, *J* = 6.8 Hz, $OC\underline{H}_2$), 2.46 (s, 3H, $COC\underline{H}_3$), 2.44-2.26 (m, 4H, $SC\underline{H}_2C\underline{H}_2$), 2.02 (s, 3H, $SC\underline{H}_3$), 1.73-1.65 (m, 2H, $OCH_2C\underline{H}_2$), 1.44-1.34 (m, 2H, $O(CH_2)_2C\underline{H}_2$), 0.90 (t, 3H, *J* = 7.6 Hz, $O(CH_2)_3C\underline{H}_3$); ^{13}C NMR (100 MHz, $CDCl_3$) 188.2 (C4), 169.8 (N$\underline{C}$=OMe), 166.8 (ester $\underline{C}$=O), 164.1 (C2), 98.4 (C3), 65.4 ($O\underline{C}H_2$), 56.9 (C5), 30.2 ($OCH_2\underline{C}H_2$), 28.4 ($S\underline{C}H_2$), 27.8 ($SCH_2\underline{C}H_2$), 25.2 ($NC{=}O\underline{C}H_3$), 18.8 ($O(CH_2)_2\underline{C}H_2$), 15.1 ($S\underline{C}H_3$), 13.5 ($O(CH_2)_3\underline{C}H_3$); MS ($ES^-$) 314.12 (M − H); HRMS (M − H), calcd for $C_{14}H_{20}N_1O_5S_1$, 314.1068; found, 314.1069.

Synthesis of **9b**

Tetramic acid **9b** was obtained from tetramic acid **8b** by using the same method as for tetramic acid **9a**; Yield 92%; mp 80 °C; ^{1}H NMR (400 MHz, $CDCl_3$) 4.87-4.85 (m, 1H, C5), 4.39-4.28 (m, 2H, $OC\underline{H}_2$), 2.43-2.30 (m, 3H), 2.06 (s, 3H, $SC\underline{H}_3$), 1.82-1.65 (m, 7H), 1.51-1.18 (m, 9H), 0.94 (t, 3H, *J* = 8.0 Hz, $O(CH_2)_3C\underline{H}_3$); ^{13}C NMR (100 MHz, $CDCl_3$); 188.5 (C4), 176.5 (N$\underline{C}$=OCH), 167.1 (ester $\underline{C}$=O), 163.9 (C2), 98.6 (C3), 65.6 ($O\underline{C}H_2$), 57.0 (C5), 44.0 (NC=O$\underline{C}$H), 30.4 (CH_2), 30.1 (CH_2), 28.6 (CH_2), 27.9 (CH_2), 27.8 (CH_2), 25.8 (CH_2), 25.2 (CH_2), 18.9 (CH_2), 15.3 ($S\underline{C}H_3$), 13.6

(O(CH_2)$_3$<u>C</u>H_3). MS (ES^-); 382.17 (M − H); HRMS (M − H), calcd for $C_{19}H_{28}N_1O_5S_1$, 382.1694; found, 382.1698.

Synthesis of 3-carboxamide tetramic acids **2** and **3**

General method: To a solution of 3-alkoxycarbonyl tetramic acid (1.0 equiv) in toluene was added primary amine (1.0 equiv), and the mixture was heated under reflux for 4 hours. After completion of the reaction as indicated by TLC, the solvent was evaporated, and column chromatography gave metal-chelated 3-carboxamide tetramic acid. The compound was dissolved in dichloromethane (50 mL) and washed with 1 N HCl (20 mL). The organic layer was dried with $MgSO_4$ and concentrated in vacuo to give pure 3-carboxamide tetramic acid.

Synthesis of **2a**

Yield 66%; mp 97 °C; Form AB:CD = 90:10; ^{1}H NMR (400 MHz, $CDCl_3$) 9.81 (brs, 1H, NH or OH), 9.55 (brs, 1H, NH or OH AB), 9.25 (brs, 1H, NH or OH AB), 7.51 (d, 2H, J = 8.8 Hz, C14 and C15 AB), 7.39 (d, 2H, J = 8.8 Hz, C14 and C15 CD), 6.91 (d, 2H, J = 8.8 Hz, C16 and C17), 3.98 (s, 2H, C5 AB), 3.87 (t, 4H, J = 4.8 Hz, C21 and C22), 3.77 (s, 2H, C5 CD), 3.43 (t, 2H, J = 7.2 Hz, C6), 3.13 (t, 4H, J = 4.8 Hz, C19 and C20), 1.59-1.54 (m, 2H, C7), 1.34-1.25 (m, 6H, C8-10), 0.89 (t, 3H, J = 6.8 Hz, C11); ^{13}C NMR (100 MHz, $CDCl_3$, only AB tautomer) 180.5 (C4), 168.8 (C2), 163.7 (C12), 148.2 (C18), 129.7 (C13), 121.4 (C14 and C15), 116.3 (C16 and C17), 99.6 (C3), 66.7 (C21 and C22), 49.7 (C5), 49.5 (C19 and C20), 41.4 (C6), 31.4 (CH_2), 28.4 (CH_2), 26.4 (CH_2), 22.5 (CH_2), 14.0 (C11); MS (ES^-) 386.22; HRMS (M − H) calcd for $C_{21}H_{28}N_3O_4$, 386.2085; found, 386.2092.

Synthesis of **2b**

Yield 90%; mp 67 °C; Form AB:CD = 90:10; ^{1}H NMR (500 MHz, $CDCl_3$) 11.10 (s, 1H, NH), 9.70 (s, 1H, OH AB), 9.30 (s, 1H, OH CD), 7.61 (d, 2H, *J* = 8.0 Hz, C14 and C15 AB), 7.51 (d, 2H, *J* = 7.6 Hz, C14 and C15 CD), 7.34 (dd, 2H, J_1 = 8.0 Hz, J_2 = 7.6 Hz, C16 and C17), 7.34 (dd, 1H, J_1 = 7.6 Hz, J_2 = 7.6 Hz, C18), 3.97 (s, 2H, C5 AB), 3.79 (s, 2H, C5 CD), 3.43 (t, 2H, *J* = 7.2 Hz, C6), 1.58-1.53 (m, 2H, C7), 1.36-1.26 (m, 6H, C8-10), 0.89 (t, 3H, *J* = 6.8 Hz, C11); ^{13}C NMR (125 MHz, $CDCl_3$); 188.8 (C4 CD), 180.6 (C4 AB), 175.8 (C2 CD), 168.6 (C2 AB), 165.2 (C12 CD), 164.0 (C12 AB), 136.9 (C13 AB), 136.3 (C13 CD), 129.0 (C16 and C17 CD), 128.9 (C16 and C17 AB), 124.7 (C18 CD), 124.4 (C18 AB), 120.5 (C14 and C15 CD), 120.0 (C14 and C15 AB), 99.7 (C3 AB), 87.2 (C3 CD), 55.4 (C5 CD), 49.4 (C5 AB), 42.2 (C6 CD), 41.3 (C6 AB), 31.3 (C9 AB), 31.2 (C9 CD), 28.3 (C7 AB), 27.9 (C7 CD), 26.3 (C8 AB), 26.2 (C8 CD), 22.4 (C10), 13.9 (C11); MS (ES^-) 301.1; HRMS (M + H), calcd for $C_{17}H_{23}N_2O_3$, 303.1703; found, 303.1700.

Synthesis of **2c**

Yield 88% (oil); Form AB:CD = 80:20; ^{1}H NMR (400 MHz, $CDCl_3$) 8.88 (brs, 1H, OH), 7.65 (brs, 1H, NH), 3.90 (s, 2H, C5 AB), 3.85-3.81 (m, 1H, C13), 3.67 (s, 2H, C5 CD), 3.38 (t, 2H, *J* = 7.2 Hz, C6), 1.91-1.88 (m, 2H, CH_2), 1.74-1.71 (m, 2H, CH_2), 1.60-1.52 (m, 3H, CH_2), 1.42-1.17 (m, 11H, CH_2), 0.88 (t, 3H, *J* = 6.4 Hz, C11); ^{13}C NMR (125 MHz, $CDCl_3$); 189.8 (C4 CD), 181.4 (C4 AB), 175.2 (C2 CD), 169.1 (C2 AB), 165.7 (C12 CD), 165.1 (C12 AB), 98.4 (C3 AB), 85.4 (C3 CD), 55.0 (C5 CD), 49.8 (C5 AB), 48.9 (C13 CD), 47.3 (C13 AB), 41.5 (C6 CD), 41.3 (C6 AB), 32.8 (CH_2), 32.7 (CH_2), 31.4 (CH_2), 29.6 (CH_2), 28.4 (CH_2), 28.0 (CH_2), 26.4 (CH_2), 26.3

(CH_2), 25.4 (CH_2), 25.1 (CH_2), 24.5 (CH_2), 22.5 (CH_2), 13.9 (C11); MS (ES^-) 307.2 (M − H); HRMS (M + Na), calcd for $C_{17}H_{28}N_2Na_1O_3$, 331.1992; found, 331.1993.

Synthesis of compound **2d**

Yield 41% (oil); Form AB:CD = 80:20; ^{1}H NMR (400 MHz, $CDCl_3$) 9.14 (brs, 1H, OH), 7.70 (brs, 1H, NH), 3.90 (s, 2H, C5 AB), 3.68 (s, 2H, C5 CD), 3.39-3.16 (m, 4H, C6 and C13), 1.56-1.51 (m, 4H, C7 and C14), 1.37-1.28 (m, 12H, C8-10 and C15-17), 0.87 (t, 6H, *J* = 6.8 Hz, C11 and C18); ^{13}C NMR (125 MHz, $CDCl_3$) 189.8 (C4 CD), 181.1 (C4 AB), 175.2 (C2 CD), 169.1 (C2 AB), 166.6 (C12 AB), 166.0 (C12 AB), 98.6 (C3 AB), 85.6 (C3 CD), 55.0 (C5 CD), 49.7 (C5 AB), 41.5 (CH_2), 41.2 (CH_2), 39.6 (CH_2), 38.4 (CH_2), 31.4 (CH_2), 31.3 (CH_2), 29.4 (CH_2), 28.4 (CH_2), 28.0 (CH_2), 26.5 (CH_2), 26.4 (CH_2), 22.5 (CH_2), 13.9 (C11 and C18); MS (ES^-) 309.2; HRMS (M − H), calcd for $C_{17}H_{29}N_2O_3$, 309.2184; found, 309.2178.

Synthesis of **2e**

Yield 58% (oil); Form AB:CD = 80:20; ^{1}H NMR (400 MHz, $CDCl_3$) 9.44 (brs, 1H, OH), 7.70 (brs, 1H, NH), 3.91 (s, 2H, C5 AB), 3.68 (s, 2H, C5 CD), 3.40-3.30 (m, 4H, C6 and C13), 1.59-1.52 (m, 4H, C7 and C14), 1.35-1.25 (m, 18H, C8-10 and C15-20), 0.89-0.85 (m, 6H, C11 and C21); ^{13}C NMR (100 MHz, $CDCl_3$, Only AB tautomer) 181.1 (C4), 169.1 (C2), 166.0 (C12), 98.6 (C3), 49.7 (C5), 41.3 (CH_2), 38.4 (CH_2), 31.8 (CH_2), 31.4 (CH_2), 29.4 (CH_2), 29.4 (CH_2), 29.2 (CH_2), 29.2 (CH_2), 28.4 (CH_2), 26.8 (CH_2), 26.4 (CH_2), 22.6 (CH_2), 22.5 (CH_2),14.1 (CH_3), 14.0 (CH_3); MS (ES^-) 351.3; HRMS (M − H) calcd for $C_{20}H_{35}N_2O_3$, 351.2653; found, 351.2657.

Synthesis of **2f**

Yield, 55% (oil); Form AB:CD = 80:20; ^{1}H NMR (400 MHz, $CDCl_3$) 7.71 (brs, 1H, NH), 3.91 (s, 2H, C5 AB), 3.69 (s, 2H, C5 CD), 3.41-3.31 (m, 4H, $NC\underline{H}_2$ and $NHC\underline{H}_2$), 1.56-1.54 (m, 4H, $NCH_2C\underline{H}_2$ and $NHCH_2C\underline{H}_2C7$), 1.30-1.25 (m, 24H, $C\underline{H}_2$), 0.90-0.86 (m, 6H, $C\underline{H}_3$); ^{13}C NMR (100 MHz, $CDCl_3$, AB tautomer only) 181.09 (C4), 169.09 (C2), 165.98 ($\underline{C}$=ONH), 98.64 (C3), 49.75 (C5), 41.28 (CH_2), 38.41 (CH_2), 31.88 (CH_2), 31.42 (CH_2), 29.58 (CH_2), 29.47 (CH_2), 29.31 (CH_2), 29.23 (CH_2), 28.40 (CH_2), 26.85 (CH_2), 26.38 (CH_2), 22.65 (CH_2), 22.49 (CH_2),14.08 (CH_3), 13.97 (CH_3); MS (ES^-) 393.3; HRMS (M − H) calcd for $C_{23}H_{41}N_2O_3$, 393.3123; found, 393.3120.

Synthesis of **2g**

Yield 54%; mp 84 °C; Form AB:CD = 90:10; ^{1}H NMR (400 MHz, $CDCl_3$) 9.03 (brs, 1H, OH), 8.09 (brs, 1H, NH), 7.36-7.25 (m, 5H, Ar-H), 4.55 (d, 2H, J = 6.0 Hz, $NHC\underline{H}_2$), 3.94 (s, 2H, C5 AB), 3.70 (s, 2H, C5 CD), 3.39 (t, 2H, J = 7.6 Hz, $NC\underline{H}_2$), 1.56-1.53 (m, 2H, $NCH_2C\underline{H}_2$), 1.37-1.26 (m, 6H, CH_2), 0.89 (t, 3H, J = 6.4 Hz, CH_3); ^{13}C NMR (100 MHz, $CDCl_3$); 189.55 (C4 CD), 180.70 (C4 AB), 175.49 (C2 CD), 168.83 (C2 AB), 166.67 ($\underline{C}$=ONH CD), 165.89 ($\underline{C}$=ONH AB), 137.52 (Ar quart-C AB), 136.67 (Ar quart-C CD), 128.80 (Ar tert-C CD), 128.67 (Ar tert-C AB), 127.85 (Ar tert-C CD), 127.53 (Ar tert-C AB), 127.47 (Ar tert-C AB), 126.87 (Ar tert-C CD), 99.17 (C3 AB), 85.95 (C3 CD), 55.22 (C5 CD), 49.57 (C5 AB), 43.21 ($NH\underline{C}H_2$ CD), 42.23 ($NH\underline{C}H_2$ AB), 41.76 ($N\underline{C}H_2$ CD), 41.25 ($N\underline{C}H_2$ AB), 31.38 (CH_2), 28.37 (CH_2), 26.35 (CH_2), 22.47 (CH_2), 13.96 (CH_3); MS (ES^-) 315.2; HRMS (M + Na) calcd for $C_{18}H_{24}N_2Na_1O_3$, 339.1679; found, 339.1674.

Synthesis of **2h**

Yield 60%; mp 96 °C; Form AB:CD = 90:10; ^{1}H NMR (400 MHz, $CDCl_3$) 9.55 (brs, 1H, NH or OH AB), 9.25 (brs, 1H, NH or OH CD), 8.27 (brs, 1H, NH or OH), 7.50 (d, 2H, *J* = 8.8 Hz, Ar-H AB), 7.38 (d, 2H, *J* = 8.8 Hz, Ar-H CD), 6.89 (d, 2H, *J* = 8.8 Hz, Ar-H), 5.08 (t, 1H, *J* = 6.8 Hz, $C\underline{H}{=}C$), 3.96 (s, 2H, C5 AB), 3.86 (t, 4H, *J* = 4.8 Hz, $C\underline{H}_2OC\underline{H}_2$), 3.76 (s, 2H, C5 CD), 3.54-3.39 (m, 2H, NCH_2), 3.13 (t, 4H, *J* = 4.8 Hz, $C\underline{H}_2NC\underline{H}_2$), 2.07-1.91 (m, 2H, $CHC\underline{H}_2$), 1.68 (s, 3H, $C(C\underline{H}_3)_2$), 1.60 (s, 3H, $C(C\underline{H}_3)_2$), 1.64-1.17 (m, 5H, $C\underline{H}_2C\underline{H}(Me)C\underline{H}_2$), 0.96 (d, 3H, *J* = 6.4 Hz, $CH(C\underline{H}_3)$); ^{13}C NMR (100 MHz, $CDCl_3$, AB tautomer only) 180.52 (C4), 168.73 (C2), 163.67 ($\underline{C}{=}ONH$), 148.29 (Ar quart-C), 131.44 ($CH{=}\underline{C}(Me)_2$), 129.56 (Ar quart-C), 124.39 ($\underline{C}H{=}C(Me)_2$), 121.36 (Ar tert-C), 116.17 (Ar tert-C), 99.63 (C3), 66.78 ($\underline{C}H_2O\underline{C}H_2$), 49.56 (C5), 49.45 ($\underline{C}H_2N\underline{C}H_2$), 39.43 ($N\underline{C}H_2$), 36.81 ($NCH_2CH_2CH(Me)\underline{C}H_2$), 35.23 ($NCH_2\underline{C}H_2$), 29.98 ($CH_2\underline{C}H(Me)CH_2$), 25.64 ($C(\underline{C}H_3)_2$), 25.29 ($CH\underline{C}H_2$), 19.27 ($CH(\underline{C}H_3)$), 17.62 ($C(\underline{C}H_3)_2$); MS ($ES^-$) 440.29 (M − H); HRMS (M − H) calcd for $C_{25}H_{34}N_3O_4$, 440.2555; found, 440.2553.

Synthesis of **3a**

Yield 45% (oil); AB:CD = 85:15; ^{1}H NMR (400 MHz, $CDCl_3$) 11.85 (brs, 1H, OH), 8.60 (brs, 1H, NH CD), 8.04 (brs, 1H, NH AB), 4.73 (t, 1H, *J* = 4.0 Hz, C5 AB), 4.41 (brs, 1H, C5 CD), 3.63-3.43 (m, 6H, cy-Hx $C\underline{H}$, $NHC\underline{H}_2$, $OC\underline{H}_2$ and $OC\underline{H}$), 2.46-2.34 (m, 2H, $SC\underline{H}_2$), 2.28-2.21 (m, 2H, $SCH_2C\underline{H}_2$), 2.04 (s, 3H, $SC\underline{H}_3$), 1.93-1.65 (m, 7H, $NHCH_2C\underline{H}_2$ and cy-Hx CH_2), 1.49-1.22 (m, 5H, cy-Hx CH_2), 1.18 (d, 6H, *J* = 6.0 Hz, $CH(C\underline{H}_3)_2$). ^{13}C NMR (100 MHz, $CDCl_3$, AB tautomer only); 189.4 (C4), 176.0 ($N\underline{C}{=}O$), 167.3 (C2), 166.0 ($\underline{C}{=}ONH$), 95.4 (C3), 72.0 ($O\underline{C}H$), 66.6 ($O\underline{C}H_2$), 58.6 (C5), 44.1 (cy-Hx $\underline{C}H$), 37.7 ($NH\underline{C}H_2$), 30.0 (CH_2), 29.0 (CH_2), 28.5 (CH_2), 28.0

(CH_2), 27.9 (CH_2), 25.8 (CH_2), 25.7 (CH_2), 25.4 (CH_2), 21.8 (CH(<u>C</u>H_3)$_2$), 15.2 (S<u>C</u>H_3); MS (ES^-) 425.21 (M − H); HRMS (M − H) calcd for $C_{21}H_{33}N_2O_5S_1$, 425.2116; found, 425.2118.

Synthesis of **3b**

Yield 52%; mp 97 °C; AB:CD = 85:15; ^{1}H NMR (400 MHz, $CDCl_3$) 11.96 (brs, 1H, OH), 8.31 (d, 1H, *J* = 8.0 Hz, NH CD), 7.46 (d, 1H, *J* = 8.0 Hz, NH AB), 4.75 (t, 1H, *J* = 5.2 Hz, C5 AB), 4.44 (t, 1H, *J* = 5.2 Hz, C5 CD), 3.88-3.80 (m, 1H, NHC<u>H</u>), 3.49-3.44 (m, 1H, cy-Hx C<u>H</u>), 2.47-2.36 (m, 2H, SC<u>H</u>$_2$), 2.29-2.20 (m, 2H, SCH_2C<u>H</u>$_2$), 2.05 (s, 3H, SC<u>H</u>$_3$ AB), 2.04 (s, 3H, SC<u>H</u>$_3$ CD), 1.94-1.60 (m, 10H, CH_2), 1.50-1.23 (m, 10H, CH_2); ^{13}C NMR (100 MHz, $CDCl_3$); 192.0 (C4 CD), 189.6 (C4 AB), 176.0 (N<u>C</u>=O AB), 175.7 (N<u>C</u>=O CD), 173.9 (C2 CD), 167.7 (C2 AB), 165.9 (<u>C</u>=ONH CD), 165.4 (<u>C</u>=ONH AB), 95.3 (C3 AB), 86.1 (C3 CD), 62.6 (C5 CD), 58.8 (C5 AB), 49.6 (NH<u>C</u>H CD), 48.1 (NH<u>C</u>H AB), 44.4 (cy-Hx <u>C</u>H CD), 44.1 (cy-Hx <u>C</u>H AB), 32.7 (CH_2 AB), 32.6 (CH_2 AB), 32.4 (CH_2 CD), 30.2 (CH_2 CD), 30.1 (CH_2 AB), 29.6 (CH_2 CD), 28.9 (CH_2 CD), 28.6 (CH_2 AB), 28.3 (CH_2 CD), 28.1 (CH_2 CD), 28.0 (CH_2 AB), 27.9 (CH_2 AB), 25.8 (CH_2), 25.7 (CH_2), 25.4 (CH_2 CD), 25.3 (CH_2 AB), 25.2 (CH_2 AB), 24.9 (CH_2 CD), 24.5 (CH_2 AB), 24.2 (CH_2 CD), 15.2 (S<u>C</u>H_3); MS (ES^-) 407.20 (M − H); HRMS (M − H) calcd for $C_{21}H_{31}N_2O_4S_1$, 407.2010; found, 407.2012.

Synthesis of **3c**

Yield, 64%; mp 46 °C; AB:CD = 80:20; ^{1}H NMR (400 MHz, $CDCl_3$) 11.86 (brs, 1H, NH or OH), 8.56 (brs, 1H, NH or OH CD), 7.59 (brs, 1H, NH or OH AB), 4.72 (dd, 1H, J_1 = 6.0 Hz, J_2 = 4.0 Hz, C5 AB), 4.44 (t, 1H, *J* = 4.4 Hz, C5 CD), 2.55 (s, 3H, C=OC<u>H</u>$_3$ CD), 2.53 (s, 3H, C=OC<u>H</u>$_3$ AB), 2.51-2.41 (m, 2H, SC<u>H</u>$_2$), 2.38-2.25 (m, 2H,

$SCH_2C\underline{H}_2$), 2.08 (s, 3H, $SC\underline{H}_3$ AB), 2.06 (s, 3H, $SC\underline{H}_3$ CD),1.78 (s, 2H, $CC\underline{H}_2C$), 1.52 (s, 6H, $NHC(C\underline{H}_3)_2$ CD), 1.50 (s, 6H, $NHC(C\underline{H}_3)_2$ AB), 1.01 (s, 9H, $C(C\underline{H}_3)_3$); ^{13}C NMR (100 MHz, $CDCl_3$); 191.8 (C4 CD), 190.6 (C4 AB), 174.4 ($N\underline{C}$=O CD), 169.6 ($N\underline{C}$=O AB), 169.2 (C2 CD), 168.3 (C2 AB), 166.7 ($\underline{C}$=ONH CD),166.3 ($\underline{C}$=ONH AB), 94.7 (C3 AB), 86.4 (C3 CD), 62.6 (C5 CD), 59.0 (C5 AB), 57.7 ($NH\underline{C}(Me)_2$ CD), 56.4 ($NH\underline{C}(Me)_2$ AB), 52.4 ($C\underline{C}H_2C$ CD), 52.1 ($C\underline{C}H_2C$ AB), 31.6 ($\underline{C}(Me)_3$), 31.3 ($C(\underline{C}H_3)_3$ AB), 31.2 ($C(\underline{C}H_3)_3$ CD), 29.3 ($NHC(\underline{C}H_3)_2$ CD), 29.2 ($NHC(\underline{C}H_3)_2$ AB), 28.7 ($S\underline{C}H_2$ CD), 28.5 ($S\underline{C}H_2$ AB), 28.4 ($SCH_2\underline{C}H_2$ CD), 28.2 ($SCH_2\underline{C}H_2$ AB), 25.5 ($C=O\underline{C}H_3$ CD), 25.3 ($C=O\underline{C}H_3$ AB), 15.3 ($S\underline{C}H_3$); MS (ES^-) 369.19 (M-H); MS (ES^+) 371.21 (M + H), 393.19 (M + Na); HRMS (M + Na) calcd for $C_{18}H_{30}N_2O_4S_1$, 393.1818; found, 393.1805.

Synthesis of **3d**

Yield 58%; mp 91 °C; AB:CD = 80:20; ^1H NMR (400 MHz, $CDCl_3$) 11.15 (brs, 1H, OH), 7.87 (d, 1H, J = 6.4 Hz, NH AB), 7.34 (d, 1H, J = 6.4 Hz, NH CD), 4.76 (t, 1H, J = 5.2 Hz, C5 AB), 4.47-4.43 (m, 1H, C5 CD), 4.28-4.26 (m, 1H, $NHC\underline{H}$ AB), 4.09-3.98 (m, 1H, $NHC\underline{H}$ CD), 2.54 (s, 3H, $C=OC\underline{H}_3$ CD), 2.52 (s, 3H, $C=OC\underline{H}_3$ AB), 2.51-2.42 (m, 2H, $SC\underline{H}_2$), 2.38-2.26 (m, 2H, $SCH_2C\underline{H}_2$), 2.09 (s, 3H, $SC\underline{H}_3$ AB), 2.05 (s, 3H, $SC\underline{H}_3$ CD),2.00-1.17 (m, 6H, cy-Hx $C\underline{H}_2$), 1.08-0.93 (m, 9H, cy-Hx $C\underline{H}_3$); ^{13}C NMR (100 MHz, $CDCl_3$, diastereo mixture, tautomer AB only); 189.9 and 189.8 (C4), 169.6 and 169.5 ($N\underline{C}$=O), 168.2 and 168.1 (C2), 165.5 and 165.4 ($\underline{C}$=ONH AB), 95.0 (C3), 58.9 and 58.8 (C5), 47.4 and 47.3 (CH_2), 45.7 and 45.6 ($NH\underline{C}H$), 41.6 and 41.5 (CH_2), 38.3 and 38.2 (CH_2), 33.8 (CH_3), 30.6 ($\underline{C}(CH_3)$), 28.6 and 28.5 ($S\underline{C}H_2$), 28.2 and 28.1 ($SCH_2\underline{C}H_2$), 27.8 (CH_3), 25.4 and 25.3 ($C=O\underline{C}H_3$), 22.3 and 22.2 (CH_3) 15.3

($S\underline{C}H_3$); MS (ES^-) 381.19 (M − H), MS (ES^+), 383.20 (M + H); HRMS (M + Na) calcd for $C_{19}H_{30}N_2O_4S_1$, 405.1818; found, 405.1814.

Synthesis of **3e**

Yield 63%; mp 159 °C; AB:CD = 99:1; ^{1}H NMR (400 MHz, DMSO) 10.89 (s, 1H, NH or OH), 7.67 (d, 2H, *J* = 8.8 Hz, Ar-H), 7.43 (d, 2H, *J* = 8.8 Hz, Ar-H), 4.14 (dd, 1H, J_1 = 6.0 Hz, J_2 = 4.0 Hz, C5), 3.14 (s, 6H, $N(C\underline{H}_3)_2$), 2.42 (s, 3H, $C{=}O\underline{C}H_3$), 2.38-2.31 (m, 2H, $SC\underline{H}_2$), 2.17-2.03 (m, 2H, $SCH_2C\underline{H}_2$), 1.99 (s, 3H, $SC\underline{H}_3$); ^{13}C NMR (100 MHz, DMSO, tautomer AB only); 190.4 (C4), 170.5 ($N\underline{C}{=}O$), 168.8 (C2), 162.7 ($\underline{C}{=}ONH$), 138.8 (Ar quart-C), 125.3 (Ar quart-C), 120.0 (Ar tert-C), 119.5 (Ar tert-C), 92.9 (C3), 59.2 (C5), 45.4 ($N(\underline{C}H_3)_2$), 29.0 ($S\underline{C}H_2$), 27.7 ($SCH_2\underline{C}H_2$), 25.3 ($C{=}O\underline{C}H_3$), 14.7 ($S\underline{C}H_3$); MS (ES^-), 376.14 (M − H); HRMS (M + Na), calcd for $C_{18}H_{23}N_3O_4S_1$, 400.1301; found, 400.1290.

Synthesis of **3f**

Yield 60%; mp 170 °C; AB:CD = 99:1; ^{1}H NMR (400 MHz, $CDCl_3$) 9.21 (s, 1H, NH or OH), 8.18 (s, 1H, NH or OH), 7.48 (d, 2H, *J* = 8.0 Hz, Ar-H), 6.91 (d, 2H, *J* = 8.0 Hz, Ar-H), 4.89 (brs, 1H, C5), 3.87 (t, 4H, *J* = 4.4 Hz, $C\underline{H}_2OC\underline{H}_2$), 3.16 (t, 4H, *J* = 4.4 Hz, $C\underline{H}_2NC\underline{H}_2$), 2.58 (s, 3H, $C{=}O\underline{C}H_3$), 2.55-2.45 (m, 2H, $SC\underline{H}_2$), 2.41-2.36 (m, 2H, $SCH_2C\underline{H}_2$), 2.10 (s, 3H, $SC\underline{H}_3$); ^{13}C NMR (100 MHz, $CDCl_3$, tautomer AB only); 187.9 (C4), 169.5 ($N\underline{C}{=}O$), 165.5 (C2), 163.6 ($\underline{C}{=}ONH$), 148.9 (Ar quart-C), 128.4 (Ar quart-C), 121.8 (Ar tert-C), 116.2 (Ar tert-C), 97.6 (C3), 66.8 ($\underline{C}H_2O\underline{C}H_2$), 58.3 (C5), 49.3 ($\underline{C}H_2N\underline{C}H_2$), 28.5 ($S\underline{C}H_2$), 28.2 ($SCH_2\underline{C}H_2$), 25.4 ($C{=}O\underline{C}H_3$), 15.3 ($S\underline{C}H_3$); MS ($ES^-$); 418.15 (M − H), HRMS (M − H) calcd for $C_{20}H_{24}N_3O_5S_1$, 418.1442; found, 418.1449.

10a A TAUTOMER

```
Spartan '02 Mechanics Program: (PC/x86)
Release  115B

  Reason for exit: Successful completion
  Mechanics CPU Time : 000:00:00.3
  Mechanics Wall Time: 000:00:00.3
Spartan '02 Quantum Mechanics Program:  (PC/x86)
Release  115B

Job type: Geometry optimization.
Method: RB3LYP
Basis set: 6-31G(D)
Number of shells: 62
Number of basis functions: 183

SCF model:
 A restricted hybrid HF-DFT SCF calculation will be
 performed using Pulay DIIS extrapolation


Optimization:
       Step     Energy        Max Grad.     Max Dist.
         1   -552.5962400      0.023621      0.138404
         2   -552.6036072      0.009644      0.032444
         3   -552.6042932      0.002787      0.016188
         4   -552.6044155      0.001086      0.004488
         5   -552.6044243      0.000538      0.001014
         6   -552.6044231      0.000090      0.000316
  Reason for exit: Sucessful completion
  Quantum Mechanics Program CPU Time : 000:32:01.1
  Quantum Mechanics Program Wall Time: 000:21:27.0

Spartan '02 Properties Program:  (PC/x86)
Release  115B
   Orientation rotated by   179.999994991044     degrees!
  Reason for exit: Successful completion
  Properties Program CPU Time : 000:00:00.5
  Properties Program Wall Time: 000:00:00.6

** GEOMETRY OPTIMIZATION IN DELOCALIZED INTERNAL COORDINATES
**
   Searching for a Minimum

   Optimization Cycle:   6

                   Coordinates (Angstroms)
     ATOM             X          Y          Z
    1  N         1.553777  -0.263542   0.000000
    2  C         1.506838   1.185329   0.000000
```

```
   3  C          0.026292   1.449356   0.000000
   4  C         -0.676986   0.276958   0.000000
   5  C          0.292687  -0.845710   0.000000
   6  O          0.069098  -2.049253   0.000000
   7  O         -0.461222   2.671232   0.000000
   8  C         -2.129675   0.272786   0.000000
   9  O         -2.729402   1.365739   0.000000
  10  C         -2.881342  -1.031236   0.000000
  11  C          2.782826  -1.022101   0.000000
  12  H          1.987925   1.624171   0.887239
  13  H          1.987925   1.624171  -0.887239
  14  H         -1.459168   2.532007   0.000000
  15  H         -2.599379  -1.629735  -0.873274
  16  H         -3.955283  -0.835946   0.000000
  17  H         -2.599379  -1.629735   0.873274
  18  H          3.387361  -0.807592   0.891511
  19  H          3.387361  -0.807592  -0.891511
  20  H          2.509748  -2.079307   0.000000
  Point Group: cs   Number of degrees of freedom:  34


  Energy is  -552.604423124

 Hessian Updated using BFGS Update
  internal optimization with constraints (0)

 34 Hessian modes will be used to form the next step
  Hessian Eigenvalues:
    0.052511   0.055510   0.056417   0.070952   0.127769
0.130975
    0.150573   0.159305   0.177514   0.178309   0.204058
0.212112
    0.220593   0.228453   0.233546   0.254312   0.270679
0.281991
    0.296975   0.303096   0.305099   0.306704   0.307577
0.308449
    0.310524   0.329528   0.350899   0.382164   0.443485
0.464462
    0.542845   0.714131   0.813100   0.863274

 Minimum Search - Taking Simple RFO Step
 Searching for Lamda that Minimizes Along All modes
 Value Taken   Lamda =  -0.00000014
 Step Taken.  Stepsize is  0.000813

                       Maximum    Tolerance   Cnvgd?
        Gradient        0.000090     0.000300    YES
        Displacement     0.000316     0.001200    YES
        Energy change    0.000001     0.000001     NO
```

```
#####################################################
#  Entering anlman.exe on Sun May 06 04:19:42 2012  #
#####################################################

 Analysis of SCF Wavefunction

 Total job wall time:  1.3e+006 s
```

10a B TAUTOMER

```
Spartan '02 Mechanics Program:  (PC/x86)
Release  115B

  Reason for exit: Successful completion
  Mechanics CPU Time : 000:00:00.2
  Mechanics Wall Time: 000:00:00.2
Spartan '02 Quantum Mechanics Program:  (PC/x86)
Release  115B

Job type: Geometry optimization.
Method: RB3LYP
Basis set: 6-31G(D)
Number of shells: 62
Number of basis functions: 183

SCF model:
 A restricted hybrid HF-DFT SCF calculation will be
 performed using Pulay DIIS extrapolation


Optimization:
       Step     Energy        Max Grad.     Max Dist.
          1   -552.5991897      0.030375      0.107548
          2   -552.6074004      0.013960      0.038609
          3   -552.6083457      0.003796      0.016282
          4   -552.6084863      0.001023      0.004150
          5   -552.6084886      0.000204      0.000945
  Reason for exit: Sucessful completion
  Quantum Mechanics Program CPU Time : 000:26:48.4
  Quantum Mechanics Program Wall Time: 000:14:42.8

Spartan '02 Properties Program:  (PC/x86)
Release  115B
   Orientation rotated by   179.999994991044     degrees!
  Reason for exit: Successful completion
  Properties Program CPU Time : 000:00:00.3
  Properties Program Wall Time: 000:00:00.4


** GEOMETRY OPTIMIZATION IN DELOCALIZED INTERNAL COORDINATES
**
   Searching for a Minimum
```

Optimization Cycle: 5

ATOM		X	Y	Z
1	N	1.586310	-0.213839	0.000000
2	C	1.536102	1.237007	0.000000
3	C	0.037765	1.532582	0.000000
4	C	-0.652557	0.270741	0.000000
5	C	0.344093	-0.824735	0.000000
6	O	0.160021	-2.037515	0.000000
7	O	-0.472187	2.661860	0.000000
8	C	-2.028307	0.199699	0.000000
9	O	-2.747710	1.314009	0.000000
10	C	-2.824199	-1.061635	0.000000
11	C	2.826674	-0.955457	0.000000
12	H	2.016125	1.674619	-0.887578
13	H	2.016125	1.674619	0.887578
14	H	-2.111659	2.088108	0.000000
15	H	-3.475914	-1.081288	0.881724
16	H	-2.162637	-1.928429	0.000000
17	H	-3.475914	-1.081288	-0.881724
18	H	3.426266	-0.725716	-0.890975
19	H	3.426266	-0.725716	0.890975
20	H	2.575339	-2.017626	0.000000

Coordinates (Angstroms)

Point Group: cs Number of degrees of freedom: 34

Energy is -552.608488624

Hessian Updated using BFGS Update
internal optimization with constraints (0)

34 Hessian modes will be used to form the next step
Hessian Eigenvalues:
0.053121 0.056099 0.056598 0.071180 0.123833
0.132122
0.142224 0.146835 0.168981 0.182364 0.197102
0.209779
0.210467 0.221605 0.231890 0.250398 0.270173
0.273158
0.276082 0.292419 0.303718 0.306650 0.307709
0.310569
0.312854 0.321230 0.341594 0.395529 0.431717
0.512594
0.648731 0.680669 0.821649 0.866505

Minimum Search - Taking Simple RFO Step
Searching for Lamda that Minimizes Along All modes
Value Taken Lamda = -0.00000076
Step Taken. Stepsize is 0.001862

```
                 Maximum    Tolerance   Cnvgd?
Gradient         0.000204     0.000300    YES
Displacement      0.000945     0.001200    YES
Energy change    -0.000002     0.000001     NO
```

```
#####################################################
#  Entering anlman.exe on Sun May 06 04:38:49 2012  #
#####################################################

 Analysis of SCF Wavefunction

 Total job wall time:  8.8e+005 s
```

10a C TAUTOMER

```
Spartan '02 Mechanics Program:  (PC/x86)
Release  115B

  Reason for exit: Successful completion
  Mechanics CPU Time : 000:00:00.1
  Mechanics Wall Time: 000:00:00.2
Spartan '02 Quantum Mechanics Program:  (PC/x86)
Release  115B

Job type: Geometry optimization.
Method: RB3LYP
Basis set: 6-31G(D)
Number of shells: 62
Number of basis functions: 183

SCF model:
 A restricted hybrid HF-DFT SCF calculation will be
 performed using Pulay DIIS extrapolation


Optimization:
       Step     Energy        Max Grad.     Max Dist.
         1    -552.5871228     0.049436      0.130259   1
         2    -552.6007876     0.010505      0.187450   1
         3    -552.6026218     0.005646      0.204341   1
         4    -552.6030211     0.003783      0.161886   1
         5    -552.6029772     0.006357      0.222468
         6    -552.6034428     0.003213      0.211975
         7    -552.6037099     0.001543      0.230797
         8    -552.6038365     0.000878      0.169330
         9    -552.6038745     0.001926      0.038243
        10    -552.6039220     0.000481      0.048647
        11    -552.6039304     0.001018      0.020277
        12    -552.6039486     0.000291      0.023252
```

```
        13   -552.6039530      0.000141      0.022391
        14   -552.6039503      0.000092      0.041845
        15   -552.6039492      0.000197      0.177355
        16   -552.6039341      0.000668      0.142921   1
        17   -552.6032730      0.002955      0.091064
        18   -552.6039558      0.000396      0.183535
        19   -552.6040081      0.000446      0.187290
        20   -552.6040519      0.000825      0.186118
        21   -552.6040740      0.001373      0.168282
        22   -552.6041266      0.001100      0.178649
        23   -552.6041548      0.000841      0.135319
        24   -552.6041878      0.000604      0.141565
        25   -552.6041838      0.000745      0.016972
        26   -552.6041962      0.000174      0.045826
        27   -552.6041956      0.000051      0.064522
  Reason for exit: Sucessful completion
  Quantum Mechanics Program CPU Time : 002:43:15.1
  Quantum Mechanics Program Wall Time: 001:37:27.3

Spartan '02 Properties Program:  (PC/x86)
Release  115B
  Reason for exit: Successful completion
  Properties Program CPU Time : 000:00:00.5
  Properties Program Wall Time: 000:00:01.2


** GEOMETRY OPTIMIZATION IN DELOCALIZED INTERNAL COORDINATES
**
   Searching for a Minimum

   Optimization Cycle:  27

                    Coordinates (Angstroms)
     ATOM              X           Y           Z
    1  N         1.622430    0.015427    0.036597
    2  C         1.238259    1.416276    0.206894
    3  C        -0.318129    1.394335    0.173928
    4  C        -0.680089    0.005968   -0.013348
    5  C         0.521615   -0.740254   -0.082995
    6  O         0.583479   -2.035572   -0.246280
    7  O        -1.000475    2.402077    0.293156
    8  C        -1.931839   -0.679531   -0.128346
    9  O        -1.944897   -1.928625   -0.285233
   10  C        -3.231414    0.082256   -0.064282
   11  C         2.990364   -0.461686    0.007407
   12  H         1.634059    2.039612   -0.603918
   13  H         1.600486    1.816011    1.161582
   14  H        -0.415060   -2.304435   -0.299995
   15  H        -3.794957   -0.095132   -0.987427
   16  H        -3.836403   -0.311895    0.760409
   17  H        -3.065075    1.152163    0.073285
```

```
  18  H         3.540961  -0.005807  -0.824335
  19  H         3.504271  -0.216260   0.944595
  20  H         2.982415  -1.544929  -0.121693
  Point Group: c1   Number of degrees of freedom:  54


  Energy is  -552.604195637

 Hessian Updated using BFGS Update
  internal optimization with constraints (0)

 54 Hessian modes will be used to form the next step
  Hessian Eigenvalues:
    0.000187   0.000846   0.002519   0.006532   0.011875
0.019201
    0.021012   0.023297   0.033718   0.035985   0.052266
0.054367
    0.055015   0.055549   0.056147   0.063491   0.066917
0.089166
    0.107864   0.128599   0.131212   0.138485   0.151339
0.163736
    0.167491   0.185045   0.198706   0.212166   0.215742
0.228407
    0.229589   0.239898   0.253628   0.263287   0.270964
0.294017
    0.303656   0.305190   0.305775   0.307706   0.307857
0.308582
    0.310140   0.312733   0.327645   0.338045   0.389876
0.410328
    0.422808   0.470423   0.546972   0.755577   0.841212
0.864885

 Minimum Search - Taking Simple RFO Step
 Searching for Lamda that Minimizes Along All modes
 Value Taken   Lamda =  -0.00000218
 Step Taken.  Stepsize is  0.102556

                        Maximum    Tolerance   Cnvgd?
        Gradient         0.000051     0.000300    YES
        Displacement      0.064522     0.001200     NO
        Energy change     0.000001     0.000001    YES


#####################################################
#  Entering anlman.exe on Sun May 06 06:19:46 2012  #
#####################################################

 Analysis of SCF Wavefunction

 Total job wall time:  5.8e+006 s
```

10a D TAUTOMER

```
Spartan '02 Mechanics Program:  (PC/x86)
Release  115B

  Reason for exit: Successful completion
  Mechanics CPU Time : 000:00:00.1
  Mechanics Wall Time: 000:00:00.3
Spartan '02 Quantum Mechanics Program:  (PC/x86)
Release  115B

Job type: Geometry optimization.
Method: RB3LYP
Basis set: 6-31G(D)
Number of shells: 62
Number of basis functions: 183

SCF model:
 A restricted hybrid HF-DFT SCF calculation will be
 performed using Pulay DIIS extrapolation


Optimization:
       Step     Energy        Max Grad.     Max Dist.
         1   -552.6004501      0.031675      0.103239
         2   -552.6096787      0.014116      0.164904
         3   -552.6107933      0.004552      0.171504
         4   -552.6108469      0.002244      0.175373
         5   -552.6109721      0.002005      0.066418
         6   -552.6110430      0.000578      0.022524
         7   -552.6110482      0.000450      0.012837
         8   -552.6110527      0.000170      0.005036
         9   -552.6110516      0.000048      0.006513
        10   -552.6110480      0.000033      0.003052
        11   -552.6110489      0.000023      0.000682
  Reason for exit: Sucessful completion
  Quantum Mechanics Program CPU Time : 001:02:06.8
  Quantum Mechanics Program Wall Time: 000:43:51.0

Spartan '02 Properties Program:  (PC/x86)
Release  115B
  Reason for exit: Successful completion
  Properties Program CPU Time : 000:00:00.4
  Properties Program Wall Time: 000:00:00.8


** GEOMETRY OPTIMIZATION IN DELOCALIZED INTERNAL COORDINATES
**
   Searching for a Minimum
```

```
 Optimization Cycle:  11

                        Coordinates (Angstroms)
     ATOM              X           Y           Z
      1  N         1.652107   -0.056066    0.004685
      2  C         1.319486    1.361033    0.022632
      3  C        -0.226597    1.392437    0.018981
      4  C        -0.647364   -0.000342   -0.001899
      5  C         0.545921   -0.853051   -0.009599
      6  O         0.547159   -2.099617   -0.026313
      7  O        -0.880622    2.424147    0.032311
      8  C        -1.907677   -0.554670   -0.013741
      9  O        -2.060370   -1.869668   -0.032261
     10  C        -3.179119    0.226870   -0.007635
     11  C         3.007650   -0.561340    0.001903
     12  H         1.713012    1.887466   -0.857553
     13  H         1.708407    1.863900    0.918473
     14  H        -1.137392   -2.276755   -0.034446
     15  H        -2.972802    1.297511    0.006747
     16  H        -3.770460   -0.030844   -0.894325
     17  H        -3.775646   -0.053201    0.868767
     18  H         3.549909   -0.234202    0.898274
     19  H         3.554638   -0.212033   -0.883174
     20  H         2.959762   -1.651575   -0.011828
 Point Group: c1    Number of degrees of freedom:  54


 Energy is   -552.611048871

Hessian Updated using BFGS Update
 internal optimization with constraints (0)

54 Hessian modes will be used to form the next step
 Hessian Eigenvalues:
     0.001079    0.001422    0.005573    0.006824    0.010373
0.015754
     0.021829    0.024291    0.028709    0.043085    0.051379
0.054470
     0.055225    0.055876    0.056230    0.057753    0.072077
0.084283
     0.093563    0.126088    0.131003    0.145621    0.146875
0.151080
     0.169409    0.184129    0.192062    0.197186    0.209784
0.217475
     0.221151    0.236225    0.237880    0.264926    0.273176
0.283661
     0.293566    0.303092    0.304261    0.306800    0.307835
0.309369
     0.310416    0.311685    0.312857    0.324863    0.340515
0.388067
     0.417079    0.516116    0.570588    0.696533    0.841361
```

```
0.849564

 Minimum Search - Taking Simple RFO Step
 Searching for Lamda that Minimizes Along All modes
 Value Taken    Lamda =  -0.00000001
 Step Taken.  Stepsize is  0.001249

                      Maximum     Tolerance    Cnvgd?
        Gradient        0.000023      0.000300     YES
        Displacement     0.000682      0.001200     YES
        Energy change    -0.000001      0.000001     YES


#####################################################
#  Entering anlman.exe on Sun May 06 07:07:50 2012  #
#####################################################

 Analysis of SCF Wavefunction

 Total job wall time:  2.6e+006 s
```

11a A TAUTOMER

```
Spartan '02 Mechanics Program:  (PC/x86)
Release  115B

  Reason for exit: Successful completion
  Mechanics CPU Time : 000:00:00.2
  Mechanics Wall Time: 000:00:00.2
Spartan '02 Quantum Mechanics Program:  (PC/x86)
Release  115B
  To use a standard Psuedopotential a heavy atom must exists
  (An atom larger than 'Ar')

Job type: Geometry optimization.
Method: RB3LYP
Basis set: LACVP*
Number of shells: 92
Number of basis functions: 200

SCF model:
 A restricted hybrid HF-DFT SCF calculation will be
 performed using Pulay DIIS extrapolation


Optimization:
        Step      Energy         Max Grad.      Max Dist.
          1    -607.9706244      0.025132      0.108364
          2    -607.9780656      0.009302      0.029426
          3    -607.9787417      0.002776      0.015587
```

```
          4   -607.9788546      0.000895      0.003513
          5   -607.9788625      0.000408      0.001042
          6   -607.9788641      0.000074      0.000166
  Reason for exit: Sucessful completion
  Quantum Mechanics Program CPU Time : 000:27:32.4
  Quantum Mechanics Program Wall Time: 000:27:57.5

Spartan '02 Properties Program:  (PC/x86)
Release  115B
   Orientation rotated by   179.999994991044     degrees!
  Reason for exit: Successful completion
  Properties Program CPU Time : 000:00:00.5
  Properties Program Wall Time: 000:00:00.6


** GEOMETRY OPTIMIZATION IN DELOCALIZED INTERNAL COORDINATES
**
   Searching for a Minimum

   Optimization Cycle:   6

                    Coordinates (Angstroms)
     ATOM              X           Y           Z
    1  N          2.003578   -0.382055    0.000000
    2  C          2.116756    1.067930    0.000000
    3  C          0.672738    1.497694    0.000000
    4  C         -0.141747    0.411441    0.000000
    5  C          0.686794   -0.804587    0.000000
    6  O          0.304214   -1.975857    0.000000
    7  O          0.311315    2.768021    0.000000
    8  C         -1.597962    0.541626    0.000000
    9  O         -2.120288    1.680399    0.000000
   10  N         -2.306452   -0.604420    0.000000
   11  C          3.137000   -1.277337    0.000000
   12  C         -3.758194   -0.620521    0.000000
   13  H          2.646269    1.444047    0.887487
   14  H          2.646269    1.444047   -0.887487
   15  H         -0.694565    2.728612    0.000000
   16  H         -1.766323   -1.467083    0.000000
   17  H          3.761936   -1.133819    0.891436
   18  H          2.745511   -2.296626    0.000000
   19  H          3.761936   -1.133819   -0.891436
   20  H         -4.156683   -0.114352   -0.885978
   21  H         -4.095419   -1.658987    0.000000
   22  H         -4.156683   -0.114352    0.885978
   Point Group: cs   Number of degrees of freedom:  38


   Energy is   -607.978864132

 Hessian Updated using BFGS Update
```

```
  internal optimization with constraints (0)

 38 Hessian modes will be used to form the next step
  Hessian Eigenvalues:
     0.053074   0.054479   0.056089   0.071223   0.130382
0.131687
     0.143378   0.159769   0.165635   0.175288   0.182345
0.197909
     0.205124   0.210331   0.220329   0.229939   0.250684
0.260765
     0.271643   0.293684   0.298213   0.306600   0.307186
0.307528
     0.308327   0.309303   0.322285   0.331283   0.352002
0.382227
     0.404947   0.419200   0.443702   0.485579   0.538339
0.675581
     0.800994   0.851060

 Minimum Search - Taking Simple RFO Step
 Searching for Lamda that Minimizes Along All modes
 Value Taken    Lamda =  -0.00000005
 Step Taken.  Stepsize is  0.000442

                        Maximum    Tolerance   Cnvgd?
        Gradient         0.000074     0.000300    YES
        Displacement      0.000166     0.001200    YES
        Energy change    -0.000002     0.000001     NO


#####################################################
#  Entering anlman.exe on Sat May 19 20:40:31 2012  #
#####################################################

 Analysis of SCF Wavefunction

 Total job wall time:  1.7e+006 s
```

11a B TAUTOMER

```
Spartan '02 Mechanics Program:  (PC/x86)
Release  115B

  Reason for exit: Successful completion
  Mechanics CPU Time : 000:00:00.1
  Mechanics Wall Time: 000:00:00.1
Spartan '02 Quantum Mechanics Program:  (PC/x86)
Release  115B
  To use a standard Psuedopotential a heavy atom must exists
  (An atom larger than 'Ar')

Job type: Geometry optimization.
```

```
Method: RB3LYP
Basis set: LACVP*
Number of shells: 92
Number of basis functions: 200

SCF model:
 A restricted hybrid HF-DFT SCF calculation will be
 performed using Pulay DIIS extrapolation


Optimization:
       Step     Energy        Max Grad.     Max Dist.
         1   -607.9634195      0.042025      0.119293
         2   -607.9757193      0.012383      0.037746
         3   -607.9771848      0.004399      0.016434
         4   -607.9773666      0.001623      0.004735
         5   -607.9773829      0.000515      0.001581
         6   -607.9773838      0.000163      0.000470
  Reason for exit: Sucessful completion
  Quantum Mechanics Program CPU Time : 000:33:51.9
  Quantum Mechanics Program Wall Time: 000:34:14.4

Spartan '02 Properties Program:  (PC/x86)
Release  115B
   Orientation rotated by   179.999994991044     degrees!
  Reason for exit: Successful completion
  Properties Program CPU Time : 000:00:00.4
  Properties Program Wall Time: 000:00:00.6


** GEOMETRY OPTIMIZATION IN DELOCALIZED INTERNAL COORDINATES
**
   Searching for a Minimum

   Optimization Cycle:   6

                   Coordinates (Angstroms)
    ATOM            X          Y          Z
     1  N        2.030824  -0.323556   0.000000
     2  C        2.140826   1.130414   0.000000
     3  C        0.678628   1.598519   0.000000
     4  C       -0.113707   0.429295   0.000000
     5  C        0.721211  -0.761873   0.000000
     6  O        0.347949  -1.942514   0.000000
     7  O        0.281703   2.780893   0.000000
     8  C       -1.512542   0.470536   0.000000
     9  O       -2.138252   1.638821   0.000000
    10  N       -2.252125  -0.636911   0.000000
    11  C        3.169764  -1.210650   0.000000
    12  C       -3.706217  -0.658161   0.000000
    13  H        2.668912   1.506016  -0.887804
```

```
  14  H         2.668912   1.506016   0.887804
  15  H        -1.416053   2.342721   0.000000
  16  H        -1.700091  -1.496318   0.000000
  17  H         3.794592  -1.059636   0.890734
  18  H         2.789939  -2.234365   0.000000
  19  H         3.794592  -1.059636  -0.890734
  20  H        -4.108657  -0.159942   0.888188
  21  H        -4.108657  -0.159942  -0.888188
  22  H        -4.031551  -1.699728   0.000000
  Point Group: cs   Number of degrees of freedom:  38


  Energy is   -607.977383841

 Hessian Updated using BFGS Update
  internal optimization with constraints (0)

 38 Hessian modes will be used to form the next step
  Hessian Eigenvalues:
     0.052727   0.054623   0.056162   0.070829   0.129217
0.131888
     0.133454   0.149834   0.160627   0.169744   0.185462
0.199528
     0.208465   0.213324   0.216744   0.225666   0.239980
0.251028
     0.270389   0.285908   0.291870   0.304578   0.305262
0.306318
     0.307035   0.308725   0.311415   0.334081   0.354335
0.374813
     0.388270   0.419292   0.422847   0.542491   0.577885
0.640683
     0.824536   0.848724

 Minimum Search - Taking Simple RFO Step
 Searching for Lamda that Minimizes Along All modes
 Value Taken    Lamda =  -0.00000034
 Step Taken.  Stepsize is  0.001242

                      Maximum    Tolerance   Cnvgd?
       Gradient        0.000163     0.000300    YES
       Displacement     0.000470     0.001200    YES
       Energy change   -0.000001     0.000001    YES


#####################################################
#  Entering anlman.exe on Mon May 21 04:39:33 2012  #
#####################################################

 Analysis of SCF Wavefunction

 Total job wall time:  2.1e+006 s
```

11a C TAUTOMER

```
Spartan '02 Mechanics Program:  (PC/x86)
Release  115B

  Reason for exit: Successful completion
  Mechanics CPU Time : 000:00:00.1
  Mechanics Wall Time: 000:00:00.1
Spartan '02 Quantum Mechanics Program:  (PC/x86)
Release  115B
  To use a standard Psuedopotential a heavy atom must exists
  (An atom larger than 'Ar')

Job type: Geometry optimization.
Method: RB3LYP
Basis set: LACVP*
Number of shells: 92
Number of basis functions: 200

SCF model:
 A restricted hybrid HF-DFT SCF calculation will be
 performed using Pulay DIIS extrapolation

Optimization:
         Step      Energy          Max Grad.      Max Dist.
           1    -607.9582059      0.047656       0.118759   2
           2    -607.9719615      0.010918       0.147967   2
           3    -607.9734362      0.006198       0.168650   2
           4    -607.9739292      0.003675       0.168116   2
           5    -607.9740010      0.002312       0.202365   1
           6    -607.9743363      0.001267       0.176407   1
           7    -607.9745321      0.001624       0.162645   1
           8    -607.9746194      0.001446       0.197190
           9    -607.9747249      0.001185       0.154816
          10    -607.9747570      0.000940       0.091768
          11    -607.9747739      0.000801       0.130265
          12    -607.9748132      0.001177       0.159244   1
          13    -607.9682148      0.029147       0.124393
          14    -607.9747971      0.001768       0.168932
          15    -607.9749115      0.001096       0.167406
          16    -607.9749838      0.000839       0.186742
          17    -607.9750584      0.000548       0.198997
          18    -607.9751276      0.000746       0.207746
          19    -607.9751748      0.000681       0.213777
          20    -607.9752388      0.001056       0.170694
          21    -607.9752723      0.000729       0.072714
          22    -607.9752754      0.000541       0.023168
          23    -607.9752816      0.000150       0.014099
          24    -607.9752834      0.000084       0.004780
          25    -607.9752856      0.000021       0.001262
```

```
        26   -607.9752862      0.000009      0.000502
  Reason for exit: Sucessful completion
  Quantum Mechanics Program CPU Time : 001:46:04.5
  Quantum Mechanics Program Wall Time: 001:50:46.6

Spartan '02 Properties Program:  (PC/x86)
Release  115B
  Reason for exit: Successful completion
  Properties Program CPU Time : 000:00:00.5
  Properties Program Wall Time: 000:00:00.8

** GEOMETRY OPTIMIZATION IN DELOCALIZED INTERNAL COORDINATES
**
   Searching for a Minimum

   Optimization Cycle:  26

                   Coordinates (Angstroms)
     ATOM              X          Y          Z
    1  N         2.114878  -0.015130   0.011005
    2  C         1.790908   1.401292  -0.173150
    3  C         0.237179   1.433426  -0.181704
    4  C        -0.172813   0.077290  -0.005631
    5  C         0.973151  -0.722530   0.099126
    6  O         0.969038  -2.024062   0.264666
    7  O        -0.440530   2.453237  -0.317784
    8  C        -1.489210  -0.524337   0.068488
    9  O        -1.604927  -1.769619   0.227210
   10  N        -2.541578   0.316046  -0.041188
   11  C         3.458644  -0.549930   0.068948
   12  C        -3.923772  -0.118046   0.008214
   13  H         2.195551   1.782528  -1.118473
   14  H         2.190252   2.010876   0.646434
   15  H        -0.030965  -2.247533   0.290921
   16  H        -2.309823   1.298638  -0.166784
   17  H         4.003540  -0.336620  -0.859118
   18  H         4.015267  -0.115975   0.908839
   19  H         3.400128  -1.631068   0.204424
   20  H        -3.932287  -1.201588   0.132731
   21  H        -4.452398   0.342979   0.851232
   22  H        -4.450235   0.140126  -0.918405
   Point Group: c1   Number of degrees of freedom:  60


   Energy is   -607.975286203

 Hessian Updated using BFGS Update
  internal optimization with constraints (0)

 60 Hessian modes will be used to form the next step
  Hessian Eigenvalues:
```

```
    0.000499   0.000980   0.003419   0.003729   0.005615
0.008627
    0.016833   0.019778   0.025269   0.028683   0.035951
0.047308
    0.053310   0.054458   0.054710   0.055602   0.056171
0.056844
    0.066640   0.090013   0.094680   0.131464   0.132030
0.144906
    0.162508   0.172483   0.181111   0.190039   0.197230
0.205641
    0.207097   0.212823   0.225486   0.229215   0.233188
0.253423
    0.262161   0.291884   0.301455   0.303157   0.305911
0.307876
    0.308053   0.308569   0.309598   0.315204   0.319104
0.324098
    0.348642   0.363864   0.390094   0.407800   0.429305
0.440071
    0.474413   0.500497   0.543644   0.739364   0.836474
0.845653

 Minimum Search - Taking Simple RFO Step
 Searching for Lamda that Minimizes Along All modes
 Value Taken    Lamda =   0.00000000
 Step Taken.  Stepsize is  0.001100

                       Maximum    Tolerance   Cnvgd?
        Gradient        0.000009     0.000300    YES
        Displacement     0.000502     0.001200    YES
        Energy change   -0.000001     0.000001    YES


#####################################################
#  Entering anlman.exe on Sat May 19 10:27:11 2012  #
#####################################################

 Analysis of SCF Wavefunction

 Total job wall time:  6.6e+006 s
```

11a D TAUTOMER

```
Spartan '02 Mechanics Program:  (PC/x86)
Release  115B

  Reason for exit: Successful completion
  Mechanics CPU Time : 000:00:00.3
  Mechanics Wall Time: 000:00:00.4
Spartan '02 Quantum Mechanics Program:  (PC/x86)
Release  115B
  To use a standard Psuedopotential a heavy atom must exists
```

```
  (An atom larger than 'Ar')

Job type: Geometry optimization.
Method: RB3LYP
Basis set: LACVP*
Number of shells: 92
Number of basis functions: 200

SCF model:
 A restricted hybrid HF-DFT SCF calculation will be
 performed using Pulay DIIS extrapolation


Optimization:
       Step     Energy        Max Grad.     Max Dist.
         1   -607.9639061      0.043796      0.129858   1
         2   -607.9763578      0.011497      0.046744   1
         3   -607.9778463      0.003966      0.016192
         4   -607.9780374      0.001674      0.161606   1
         5   -607.9779298      0.001686      0.143172
         6   -607.9780569      0.000427      0.013233
         7   -607.9780522      0.000155      0.008005
         8   -607.9780533      0.000090      0.004550
         9   -607.9780533      0.000061      0.008815
  Reason for exit: Sucessful completion
  Quantum Mechanics Program CPU Time : 000:39:36.5
  Quantum Mechanics Program Wall Time: 000:40:02.9

Spartan '02 Properties Program:  (PC/x86)
Release  115B
  Reason for exit: Successful completion
  Properties Program CPU Time : 000:00:00.5
  Properties Program Wall Time: 000:00:00.6


** GEOMETRY OPTIMIZATION IN DELOCALIZED INTERNAL COORDINATES
**
   Searching for a Minimum

   Optimization Cycle:   9

                   Coordinates (Angstroms)
     ATOM           X           Y           Z
    1  N         2.144713   -0.077092   -0.001004
    2  C         1.845657    1.351906    0.001563
    3  C         0.300412    1.410529    0.000491
    4  C        -0.128961    0.054546   -0.002557
    5  C         1.006763   -0.840187   -0.003226
    6  O         0.952954   -2.088951   -0.005297
    7  O        -0.378147    2.443385    0.002355
    8  C        -1.441723   -0.428591   -0.002888
```

```
   9  O         -1.679273  -1.731578  -0.004226
  10  N         -2.489368   0.396512  -0.001990
  11  C          3.482979  -0.620943   0.000343
  12  C         -3.876806  -0.039821   0.002655
  13  H          2.252535   1.857005  -0.885103
  14  H          2.251017   1.853441   0.890953
  15  H         -0.770840  -2.176607  -0.005503
  16  H         -2.238087   1.384838   0.001327
  17  H          4.039900  -0.305179   0.892589
  18  H          3.400582  -1.709654  -0.001884
  19  H          4.043003  -0.301735  -0.888702
  20  H         -4.105678  -0.621855   0.901635
  21  H         -4.512789   0.846693  -0.017264
  22  H         -4.098844  -0.656660  -0.874268
  Point Group: c1   Number of degrees of freedom:  60


  Energy is   -607.978053344

 Hessian Updated using BFGS Update
  internal optimization with constraints (0)

 60 Hessian modes will be used to form the next step
  Hessian Eigenvalues:
     0.000614   0.000910   0.004371   0.005724   0.007182
0.010917
     0.014387   0.018934   0.028600   0.031023   0.036369
0.042395
     0.051947   0.054585   0.054594   0.055619   0.056116
0.056294
     0.071804   0.091920   0.093704   0.129227   0.131554
0.137022
     0.151588   0.161480   0.165527   0.167361   0.175441
0.193342
     0.198743   0.212627   0.214247   0.216035   0.223386
0.231721
     0.240650   0.270763   0.283023   0.299460   0.304822
0.305681
     0.306159   0.306560   0.307655   0.308586   0.310129
0.310947
     0.316571   0.339660   0.351386   0.382021   0.399139
0.417850
     0.426032   0.541407   0.549274   0.655806   0.824533
0.849511

 Minimum Search - Taking Simple RFO Step
 Searching for Lamda that Minimizes Along All modes
 Value Taken    Lamda =  -0.00000028
 Step Taken.  Stepsize is  0.017131

                        Maximum    Tolerance   Cnvgd?
```

```
        Gradient          0.000061      0.000300     YES
        Displacement       0.008815      0.001200     NO
        Energy change      0.000000      0.000001     YES


#####################################################
#  Entering anlman.exe on Sat May 19 08:35:47 2012  #
#####################################################

 Analysis of SCF Wavefunction

 Total job wall time:  2.4e+006 s
```

11b A TAUTOMER

```
Spartan '02 Mechanics Program:  (PC/x86)
Release  115B

  Reason for exit: Successful completion
  Mechanics CPU Time : 000:00:00.2
  Mechanics Wall Time: 000:00:00.2
Spartan '02 Quantum Mechanics Program:  (PC/x86)
Release  115B
  To use a standard Psuedopotential a heavy atom must exists
  (An atom larger than 'Ar')

Job type: Geometry optimization.
Method: RB3LYP
Basis set: LACVP*
Number of shells: 104
Number of basis functions: 230

SCF model:
 A restricted hybrid HF-DFT SCF calculation will be
 performed using Pulay DIIS extrapolation


Optimization:
       Step      Energy        Max Grad.      Max Dist.
          1   -721.3170928      0.034108      0.114632
          2   -721.3263466      0.006482      0.028010
          3   -721.3270991      0.003467      0.014555
          4   -721.3272302      0.001268      0.005208
          5   -721.3272472      0.000500      0.001160
          6   -721.3272504      0.000069      0.000258
  Reason for exit: Sucessful completion
  Quantum Mechanics Program CPU Time : 000:36:32.4
  Quantum Mechanics Program Wall Time: 000:36:53.7

Spartan '02 Properties Program:  (PC/x86)
Release  115B
```

```
  Orientation rotated by   179.999994991044     degrees!
 Reason for exit: Successful completion
 Properties Program CPU Time : 000:00:00.6
 Properties Program Wall Time: 000:00:00.8

** GEOMETRY OPTIMIZATION IN DELOCALIZED INTERNAL COORDINATES
**
  Searching for a Minimum

  Optimization Cycle:   6

                 Coordinates (Angstroms)
    ATOM           X          Y          Z
   1  N       1.747191   0.273699   0.000000
   2  C       1.554297   1.728305   0.000000
   3  C       0.059598   1.845869   0.000000
   4  C      -0.527577   0.615156   0.000000
   5  C       0.507652  -0.412708   0.000000
   6  O       0.357682  -1.630162   0.000000
   7  O      -0.550909   3.009957   0.000000
   8  C      -1.985142   0.465578   0.000000
   9  O      -2.707391   1.489019   0.000000
  10  N      -2.475754  -0.787810   0.000000
  11  C       3.052005  -0.236863   0.000000
  12  C      -3.903850  -1.059040   0.000000
  13  C       3.224162  -1.736982   0.000000
  14  O       3.986018   0.547581   0.000000
  15  H       2.008773   2.190898   0.882998
  16  H       2.008773   2.190898  -0.882998
  17  H      -1.531769   2.770403   0.000000
  18  H      -1.796034  -1.543239   0.000000
  19  H      -4.383131  -0.629826  -0.886153
  20  H      -4.052865  -2.140384   0.000000
  21  H      -4.383131  -0.629826   0.886153
  22  H       2.748117  -2.187862   0.875264
  23  H       2.748117  -2.187862  -0.875264
  24  H       4.295168  -1.944799   0.000000
  Point Group: cs   Number of degrees of freedom:  42


  Energy is  -721.327250423

 Hessian Updated using BFGS Update
 internal optimization with constraints (0)

 42 Hessian modes will be used to form the next step
 Hessian Eigenvalues:
    0.051308   0.054479   0.055506   0.074351   0.128611
0.130299
    0.143684   0.151156   0.164729   0.166957   0.176615
0.178562
```

```
    0.198665   0.205485   0.214700   0.225257   0.232870
0.239808
    0.257597   0.267002   0.279829   0.290934   0.298472
0.305334
    0.305556   0.307019   0.307852   0.308194   0.309269
0.323222
    0.348293   0.399980   0.410308   0.419374   0.447581
0.484422
    0.543271   0.576030   0.707413   0.800702   0.807014
0.847165

 Minimum Search - Taking Simple RFO Step
 Searching for Lamda that Minimizes Along All modes
 Value Taken    Lamda =  -0.00000012
 Step Taken.  Stepsize is  0.000731

                       Maximum    Tolerance    Cnvgd?
       Gradient         0.000069     0.000300    YES
       Displacement      0.000258     0.001200    YES
       Energy change    -0.000003     0.000001     NO


#####################################################
#  Entering anlman.exe on Tue May 22 07:08:40 2012  #
#####################################################

 Analysis of SCF Wavefunction

 Total job wall time:  2.2e+006 s
```

11b B TAUTOMER

```
Spartan '02 Mechanics Program:  (PC/x86)
Release  115B

  Reason for exit: Successful completion
  Mechanics CPU Time : 000:00:00.1
  Mechanics Wall Time: 000:00:00.2
Spartan '02 Quantum Mechanics Program:  (PC/x86)
Release  115B
  To use a standard Psuedopotential a heavy atom must exists
  (An atom larger than 'Ar')

Job type: Geometry optimization.
Method: RB3LYP
Basis set: LACVP*
Number of shells: 104
Number of basis functions: 230

SCF model:
 A restricted hybrid HF-DFT SCF calculation will be
```

```
 performed using Pulay DIIS extrapolation


Optimization:
       Step     Energy        Max Grad.     Max Dist.
         1   -721.3112034      0.040058      0.121705
         2   -721.3243264      0.011609      0.042586
         3   -721.3258197      0.004764      0.013576
         4   -721.3260139      0.001838      0.005420
         5   -721.3260289      0.000613      0.001938
         6   -721.3260354      0.000189      0.000583
  Reason for exit: Sucessful completion
  Quantum Mechanics Program CPU Time : 000:49:57.5
  Quantum Mechanics Program Wall Time: 000:51:25.1

Spartan '02 Properties Program:  (PC/x86)
Release  115B
   Orientation rotated by   179.999994991044     degrees!
  Reason for exit: Successful completion
  Properties Program CPU Time : 000:00:00.7
  Properties Program Wall Time: 000:00:00.8

** GEOMETRY OPTIMIZATION IN DELOCALIZED INTERNAL COORDINATES
**
   Searching for a Minimum

   Optimization Cycle:   6

                    Coordinates (Angstroms)
     ATOM              X           Y           Z
    1  N         1.762303    0.315697    0.000000
    2  C         1.574982    1.776437    0.000000
    3  C         0.059356    1.940776    0.000000
    4  C        -0.499875    0.639054    0.000000
    5  C         0.526291   -0.373992    0.000000
    6  O         0.374300   -1.597546    0.000000
    7  O        -0.564678    3.019073    0.000000
    8  C        -1.889609    0.424753    0.000000
    9  O        -2.709602    1.460972    0.000000
   10  N        -2.423048   -0.792826    0.000000
   11  C         3.062183   -0.202027    0.000000
   12  C        -3.855111   -1.058676    0.000000
   13  C         3.230524   -1.704047    0.000000
   14  O         4.003930    0.574385    0.000000
   15  H         2.033276    2.233348   -0.883032
   16  H         2.033276    2.233348    0.883032
   17  H        -2.124795    2.286701    0.000000
   18  H        -1.737131   -1.547491    0.000000
   19  H        -4.332290   -0.632859    0.888291
   20  H        -4.332290   -0.632859   -0.888291
   21  H        -4.001018   -2.139656    0.000000
```

```
  22  H         2.753861  -2.154348   0.875092
  23  H         2.753861  -2.154348  -0.875092
  24  H         4.301305  -1.913870   0.000000
  Point Group: cs   Number of degrees of freedom:  42


  Energy is  -721.326035371

 Hessian Updated using BFGS Update
  internal optimization with constraints (0)

 42 Hessian modes will be used to form the next step
  Hessian Eigenvalues:
     0.050683   0.054613   0.056175   0.073976   0.128445
0.129220
     0.133312   0.150024   0.152524   0.160987   0.168721
0.187011
     0.200643   0.210200   0.215474   0.218866   0.233117
0.234072
     0.239895   0.255241   0.275258   0.284364   0.292792
0.304747
     0.305418   0.306020   0.306486   0.308474   0.315141
0.319688
     0.345398   0.375466   0.418989   0.422437   0.435147
0.547133
     0.605040   0.611247   0.646931   0.807260   0.824178
0.849314

 Minimum Search - Taking Simple RFO Step
 Searching for Lamda that Minimizes Along All modes
 Value Taken   Lamda =  -0.00000044
 Step Taken.  Stepsize is  0.001606

                      Maximum    Tolerance   Cnvgd?
        Gradient       0.000189     0.000300    YES
        Displacement    0.000583     0.001200    YES
        Energy change   -0.000006     0.000001     NO


#####################################################
#  Entering anlman.exe on Sun May 20 01:28:33 2012  #
#####################################################

 Analysis of SCF Wavefunction

 Total job wall time:  3.1e+006 s
```

11b C TAUTOMER

```
Spartan '02 Mechanics Program:  (PC/x86)
Release  115B
```

```
  Reason for exit: Successful completion
  Mechanics CPU Time : 000:00:00.1
  Mechanics Wall Time: 000:00:00.1
Spartan '02 Quantum Mechanics Program:  (PC/x86)
Release  115B
  To use a standard Psuedopotential a heavy atom must exists
  (An atom larger than 'Ar')

Job type: Geometry optimization.
Method: RB3LYP
Basis set: LACVP*
Number of shells: 104
Number of basis functions: 230

SCF model:
 A restricted hybrid HF-DFT SCF calculation will be
 performed using Pulay DIIS extrapolation


Optimization:
       Step     Energy        Max Grad.     Max Dist.
         1   -721.3033582      0.034912      0.089320
         2   -721.3177329      0.011315      0.040481
         3   -721.3193931      0.005967      0.019894
         4   -721.3196512      0.003336      0.010243
         5   -721.3196947      0.001995      0.002734
         6   -721.3197005      0.000395      0.001764
         7   -721.3197080      0.000145      0.000366
  Reason for exit: Sucessful completion
  Quantum Mechanics Program CPU Time : 000:54:41.0
  Quantum Mechanics Program Wall Time: 000:58:32.3

Spartan '02 Properties Program:  (PC/x86)
Release  115B
   Orientation rotated by   89.9999974955219     degrees!
  Reason for exit: Successful completion
  Properties Program CPU Time : 000:00:00.7
  Properties Program Wall Time: 000:00:00.9

** GEOMETRY OPTIMIZATION IN DELOCALIZED INTERNAL COORDINATES
**
   Searching for a Minimum

   Optimization Cycle:   7

                  Coordinates (Angstroms)
    ATOM              X          Y          Z
    1  N         1.733548   0.492020   0.000000
    2  C         1.193368   1.867492   0.000000
    3  C        -0.334164   1.668451   0.000000
```

```
  4  C        -0.547945   0.249293   0.000000
  5  C         0.681193  -0.394653   0.000000
  6  O         0.841959  -1.693861   0.000000
  7  O        -1.166364   2.573064   0.000000
  8  C        -1.777650  -0.528862   0.000000
  9  O        -1.726401  -1.787376   0.000000
 10  N        -2.932189   0.160811   0.000000
 11  C         3.124658   0.278444   0.000000
 12  C        -4.231221  -0.490669   0.000000
 13  C         3.631187  -1.145493   0.000000
 14  O         3.857376   1.250053   0.000000
 15  H         1.532964   2.412464  -0.884984
 16  H         1.532964   2.412464   0.884984
 17  H        -0.127446  -2.044552   0.000000
 18  H        -2.849994   1.174505   0.000000
 19  H        -4.353030  -1.121959  -0.886479
 20  H        -4.353030  -1.121959   0.886479
 21  H        -5.005439   0.278888   0.000000
 22  H         3.277313  -1.692487  -0.878760
 23  H         4.721027  -1.103591   0.000000
 24  H         3.277313  -1.692487   0.878760
 Point Group: cs   Number of degrees of freedom:  42


 Energy is   -721.319707975

Hessian Updated using BFGS Update
 internal optimization with constraints (0)

42 Hessian modes will be used to form the next step
 Hessian Eigenvalues:
    0.052092   0.054479   0.055874   0.067910   0.127877
0.129760
    0.139082   0.147305   0.153811   0.167254   0.187332
0.194827
    0.205191   0.210291   0.217587   0.227745   0.234326
0.243500
    0.254040   0.260418   0.269495   0.284631   0.294569
0.304611
    0.306911   0.307199   0.308508   0.309482   0.317204
0.338603
    0.356988   0.381378   0.401817   0.416051   0.435479
0.481009
    0.572756   0.714446   0.776144   0.797048   0.834008
0.883320

Minimum Search - Taking Simple RFO Step
Searching for Lamda that Minimizes Along All modes
Value Taken    Lamda =  -0.00000015
Step Taken.  Stepsize is  0.000829
```

```
                 Maximum     Tolerance    Cnvgd?
 Gradient         0.000145     0.000300     YES
 Displacement      0.000366     0.001200     YES
 Energy change    -0.000007     0.000001      NO


#####################################################
#  Entering anlman.exe on Sun May 20 00:35:24 2012  #
#####################################################

 Analysis of SCF Wavefunction

 Total job wall time:  3.5e+006 s
```

11b D TAUTOMER

```
Spartan '02 Mechanics Program:  (PC/x86)
Release  115B

  Reason for exit: Successful completion
  Mechanics CPU Time : 000:00:00.1
  Mechanics Wall Time: 000:00:00.2
Spartan '02 Quantum Mechanics Program:  (PC/x86)
Release  115B
  To use a standard Psuedopotential a heavy atom must exists
  (An atom larger than 'Ar')

Job type: Geometry optimization.
Method: RB3LYP
Basis set: LACVP*
Number of shells: 104
Number of basis functions: 230

SCF model:
 A restricted hybrid HF-DFT SCF calculation will be
 performed using Pulay DIIS extrapolation


Optimization:
       Step     Energy        Max Grad.     Max Dist.
          1   -721.3108607     0.036514      0.096415
          2   -721.3241343     0.009967      0.039701
          3   -721.3256070     0.004351      0.012127
          4   -721.3257747     0.001545      0.004641
          5   -721.3258006     0.000404      0.001221
          6   -721.3258019     0.000079      0.000449
  Reason for exit: Sucessful completion
  Quantum Mechanics Program CPU Time : 000:46:06.3
  Quantum Mechanics Program Wall Time: 000:46:38.7

Spartan '02 Properties Program:  (PC/x86)
```

```
Release  115B
   Orientation rotated by    89.9999974955219     degrees!
  Reason for exit: Successful completion
  Properties Program CPU Time : 000:00:00.7
  Properties Program Wall Time: 000:00:00.7

** GEOMETRY OPTIMIZATION IN DELOCALIZED INTERNAL COORDINATES
**
   Searching for a Minimum

   Optimization Cycle:   6

                    Coordinates (Angstroms)
     ATOM              X           Y           Z
    1  N         1.770039   0.442377   0.000000
    2  C         1.234297   1.815727   0.000000
    3  C        -0.287608   1.621661   0.000000
    4  C        -0.505706   0.211847   0.000000
    5  C         0.735346  -0.506378   0.000000
    6  O         0.864642  -1.743743   0.000000
    7  O        -1.128713   2.525541   0.000000
    8  C        -1.745564  -0.449080   0.000000
    9  O        -1.808723  -1.770923   0.000000
   10  N        -2.887226   0.231678   0.000000
   11  C         3.157220   0.242863   0.000000
   12  C        -4.209423  -0.377768   0.000000
   13  C         3.671535  -1.178336   0.000000
   14  O         3.890084   1.217765   0.000000
   15  H         1.571020   2.367120  -0.883367
   16  H         1.571020   2.367120   0.883367
   17  H        -0.857519  -2.097097   0.000000
   18  H        -2.761888   1.245929   0.000000
   19  H        -4.354981  -1.000279  -0.888544
   20  H        -4.354981  -1.000279   0.888544
   21  H        -4.951758   0.421628   0.000000
   22  H         3.313625  -1.727468   0.875398
   23  H         3.313625  -1.727468  -0.875398
   24  H         4.761638  -1.132438   0.000000
   Point Group: cs   Number of degrees of freedom:  42


   Energy is   -721.325801946

 Hessian Updated using BFGS Update
  internal optimization with constraints (0)

 42 Hessian modes will be used to form the next step
  Hessian Eigenvalues:
     0.050586   0.054578   0.055937   0.074504   0.128512
0.129191
```

```
    0.134980   0.151494   0.155212   0.161002   0.170955
0.189399
    0.201000   0.201719   0.215909   0.221731   0.232449
0.234931
    0.240054   0.256195   0.274285   0.285822   0.296150
0.304843
    0.305328   0.305593   0.306450   0.308358   0.320199
0.323266
    0.350664   0.384698   0.420613   0.424210   0.435568
0.542090
    0.570701   0.598031   0.635346   0.809012   0.830730
0.846248

 Minimum Search - Taking Simple RFO Step
 Searching for Lamda that Minimizes Along All modes
 Value Taken    Lamda =  -0.00000024
 Step Taken.  Stepsize is  0.001245

                      Maximum    Tolerance   Cnvgd?
        Gradient       0.000079     0.000300    YES
        Displacement    0.000449     0.001200    YES
        Energy change   -0.000001     0.000001     NO


#####################################################
#  Entering anlman.exe on Sat May 19 21:55:22 2012  #
#####################################################

 Analysis of SCF Wavefunction

 Total job wall time:  2.8e+006 s
```

12a A TAUTOMER

```
Spartan '02 Mechanics Program:  (PC/x86)
Release  115B

  Reason for exit: Successful completion
  Mechanics CPU Time : 000:00:00.2
  Mechanics Wall Time: 000:00:00.3
Spartan '02 Quantum Mechanics Program:  (PC/x86)
Release  115B
  To use a standard Psuedopotential a heavy atom must exists
  (An atom larger than 'Ar')

Job type: Geometry optimization.
Method: RB3LYP
Basis set: LACVP*
Number of shells: 90
Number of basis functions: 198
```

```
SCF model:
 A restricted hybrid HF-DFT SCF calculation will be
 performed using Pulay DIIS extrapolation


Optimization:
       Step     Energy        Max Grad.     Max Dist.
          1   -627.8197226      0.025385      0.116795
          2   -627.8272691      0.009268      0.031660
          3   -627.8279874      0.002559      0.012805
          4   -627.8280714      0.001132      0.003856
          5   -627.8280759      0.000537      0.001298
          6   -627.8280770      0.000073      0.000206
  Reason for exit: Sucessful completion
  Quantum Mechanics Program CPU Time : 000:33:22.7
  Quantum Mechanics Program Wall Time: 000:34:01.3

Spartan '02 Properties Program:  (PC/x86)
Release  115B
   Orientation rotated by    179.999994991044     degrees!
  Reason for exit: Successful completion
  Properties Program CPU Time : 000:00:00.4
  Properties Program Wall Time: 000:00:00.6

** GEOMETRY OPTIMIZATION IN DELOCALIZED INTERNAL COORDINATES
**
   Searching for a Minimum

   Optimization Cycle:   6

                   Coordinates (Angstroms)
     ATOM              X           Y           Z
    1  N         1.938595   -0.382245   0.000000
    2  C         1.949808    1.064872   0.000000
    3  C         0.482368    1.390134   0.000000
    4  C        -0.266097    0.250265   0.000000
    5  C         0.654339   -0.920700   0.000000
    6  O         0.394071   -2.111920   0.000000
    7  O         0.062570    2.641623   0.000000
    8  C        -1.716164    0.337180   0.000000
    9  O        -2.296610    1.432331   0.000000
   10  O        -2.363307   -0.827721   0.000000
   11  C         3.135488   -1.189525   0.000000
   12  C        -3.799367   -0.752094   0.000000
   13  H         2.447014    1.486040   0.887163
   14  H         2.447014    1.486040  -0.887163
   15  H        -0.934870    2.574812   0.000000
   16  H         3.748671   -1.000490  -0.891544
   17  H         3.748671   -1.000490   0.891544
   18  H         2.818598   -2.234445   0.000000
   19  H        -4.155826   -0.228112  -0.890570
```

```
  20  H        -4.139140  -1.787442   0.000000
  21  H        -4.155826  -0.228112   0.890570
  Point Group: cs   Number of degrees of freedom:  36


  Energy is  -627.828077004

 Hessian Updated using BFGS Update
  internal optimization with constraints (0)

 36 Hessian modes will be used to form the next step
  Hessian Eigenvalues:
    0.052994   0.056066   0.071394   0.073717   0.122326
0.130845
    0.163608   0.167895   0.174458   0.187371   0.201429
0.208767
    0.221675   0.227165   0.233493   0.257927   0.275572
0.285563
    0.288059   0.301465   0.305720   0.306454   0.307020
0.308272
    0.309420   0.333076   0.340654   0.348708   0.359480
0.400755
    0.466605   0.489248   0.522195   0.688729   0.814988
0.863556

 Minimum Search - Taking Simple RFO Step
 Searching for Lamda that Minimizes Along All modes
 Value Taken    Lamda =  -0.00000009
 Step Taken.  Stepsize is  0.000574

                       Maximum    Tolerance   Cnvgd?
       Gradient         0.000073     0.000300    YES
       Displacement      0.000206     0.001200    YES
       Energy change    -0.000001     0.000001     NO


#####################################################
#  Entering anlman.exe on Mon May 21 07:05:29 2012  #
#####################################################

 Analysis of SCF Wavefunction

 Total job wall time:  2e+006 s
```

12a B TAUTOMER

```
Spartan '02 Mechanics Program:  (PC/x86)
Release  115B

  Reason for exit: Successful completion
  Mechanics CPU Time : 000:00:00.1
```

```
  Mechanics Wall Time: 000:00:00.1
Spartan '02 Quantum Mechanics Program:  (PC/x86)
Release  115B
  To use a standard Psuedopotential a heavy atom must exists
  (An atom larger than 'Ar')

Job type: Geometry optimization.
Method: RB3LYP
Basis set: LACVP*
Number of shells: 90
Number of basis functions: 198

SCF model:
 A restricted hybrid HF-DFT SCF calculation will be
 performed using Pulay DIIS extrapolation


Optimization:
       Step     Energy        Max Grad.     Max Dist.
          1   -627.8026907      0.051123      0.144796
          2   -627.8174031      0.013123      0.053666
          3   -627.8191965      0.006469      0.019339
          4   -627.8195192      0.002610      0.011144
          5   -627.8195688      0.001111      0.003774
          6   -627.8195812      0.000265      0.001072
  Reason for exit: Sucessful completion
  Quantum Mechanics Program CPU Time : 000:33:38.9
  Quantum Mechanics Program Wall Time: 000:33:58.7

Spartan '02 Properties Program:  (PC/x86)
Release  115B
   Orientation rotated by   89.9999974955219     degrees!
  Reason for exit: Successful completion
  Properties Program CPU Time : 000:00:00.5
  Properties Program Wall Time: 000:00:00.5
** GEOMETRY OPTIMIZATION IN DELOCALIZED INTERNAL COORDINATES
**
   Searching for a Minimum

   Optimization Cycle:   6

                   Coordinates (Angstroms)
     ATOM              X           Y           Z
    1  N          1.979021   -0.321603    0.000000
    2  C          1.954366    1.128483    0.000000
    3  C          0.464571    1.449023    0.000000
    4  C         -0.239189    0.212876    0.000000
    5  C          0.718979   -0.911521    0.000000
    6  O          0.506987   -2.115050    0.000000
    7  O         -0.029667    2.596317    0.000000
    8  C         -1.628152    0.231407    0.000000
```

```
   9  O         -2.278135   1.375120   0.000000
  10  O         -2.342008  -0.872796   0.000000
  11  C          3.200949  -1.090164   0.000000
  12  C         -3.779613  -0.759885   0.000000
  13  H          2.440449   1.560351  -0.887436
  14  H          2.440449   1.560351   0.887436
  15  H         -1.563135   2.111919   0.000000
  16  H          3.807653  -0.877817   0.890970
  17  H          2.921528  -2.145585   0.000000
  18  H          3.807653  -0.877817  -0.890970
  19  H         -4.122204  -0.232683   0.893403
  20  H         -4.122204  -0.232683  -0.893403
  21  H         -4.138299  -1.788245   0.000000
  Point Group: cs   Number of degrees of freedom:  36


  Energy is   -627.819581248

 Hessian Updated using BFGS Update
  internal optimization with constraints (0)

 36 Hessian modes will be used to form the next step
  Hessian Eigenvalues:
    0.052836   0.056231   0.070661   0.075666   0.124875
0.131939
    0.134778   0.168481   0.169766   0.171682   0.195052
0.201156
    0.212630   0.222328   0.230973   0.246609   0.259947
0.266532
    0.285422   0.289791   0.304119   0.304401   0.306692
0.307523
    0.309628   0.311013   0.323544   0.364856   0.376448
0.392704
    0.474822   0.563732   0.591853   0.787348   0.849346
0.870485

 Minimum Search - Taking Simple RFO Step
 Searching for Lamda that Minimizes Along All modes
 Value Taken    Lamda =  -0.00000155
 Step Taken.  Stepsize is  0.002558

                      Maximum    Tolerance   Cnvgd?
       Gradient        0.000265     0.000300    YES
       Displacement     0.001072     0.001200    YES
       Energy change   -0.000012     0.000001     NO


#####################################################
#  Entering anlman.exe on Sun May 20 05:00:29 2012  #
#####################################################
```

Analysis of SCF Wavefunction

12a C TAUTOMER

```
Spartan '02 Mechanics Program:  (PC/x86)
Release  115B


  Reason for exit: Successful completion
  Mechanics CPU Time : 000:00:00.1
  Mechanics Wall Time: 000:00:00.2
Spartan '02 Quantum Mechanics Program:  (PC/x86)
Release  115B
  To use a standard Psuedopotential a heavy atom must exists
  (An atom larger than 'Ar')

Job type: Geometry optimization.
Method: RB3LYP
Basis set: LACVP*
Number of shells: 90
Number of basis functions: 198

SCF model:
 A restricted hybrid HF-DFT SCF calculation will be
 performed using Pulay DIIS extrapolation


Optimization:
       Step     Energy        Max Grad.     Max Dist.
          1   -627.8096298     0.043585      0.125322
          2   -627.8231459     0.011491      0.046944
          3   -627.8244373     0.004708      0.015806
          4   -627.8245822     0.004207      0.007997
          5   -627.8246200     0.000566      0.001812
          6   -627.8246243     0.000207      0.000516
  Reason for exit: Sucessful completion
  Quantum Mechanics Program CPU Time : 000:34:04.1
  Quantum Mechanics Program Wall Time: 000:34:34.8

Spartan '02 Properties Program:  (PC/x86)
Release  115B
   Orientation rotated by   89.9999974955219     degrees!
  Reason for exit: Successful completion
  Properties Program CPU Time : 000:00:00.5
  Properties Program Wall Time: 000:00:00.7

** GEOMETRY OPTIMIZATION IN DELOCALIZED INTERNAL COORDINATES
**
   Searching for a Minimum

   Optimization Cycle:   6
```

```
                    Coordinates (Angstroms)
    ATOM              X           Y           Z
   1  N         1.972316   -0.003160    0.000000
   2  C         1.742298    1.441832    0.000000
   3  C         0.189638    1.587075    0.000000
   4  C        -0.315626    0.229497    0.000000
   5  C         0.792649   -0.638905    0.000000
   6  O         0.747034   -1.953154    0.000000
   7  O        -0.375085    2.667223    0.000000
   8  C        -1.634156   -0.342024    0.000000
   9  O        -1.822400   -1.574865    0.000000
  10  O        -2.644237    0.533476    0.000000
  11  C         3.268885   -0.659275    0.000000
  12  C        -3.965340   -0.030009    0.000000
  13  H         2.170857    1.915737   -0.890658
  14  H         2.170857    1.915737    0.890658
  15  H        -0.242358   -2.161809    0.000000
  16  H         3.394104   -1.286976   -0.889132
  17  H         4.043813    0.109484    0.000000
  18  H         3.394104   -1.286976    0.889132
  19  H        -4.640371    0.825957    0.000000
  20  H        -4.123492   -0.644433   -0.890284
  21  H        -4.123492   -0.644433    0.890284
  Point Group: cs   Number of degrees of freedom:  36


  Energy is   -627.824624343

 Hessian Updated using BFGS Update
  internal optimization with constraints (0)

 36 Hessian modes will be used to form the next step
  Hessian Eigenvalues:
     0.052539   0.055287   0.067300   0.073355   0.122118
0.130548
     0.163856   0.169524   0.170162   0.178110   0.187015
0.208028
     0.225261   0.227551   0.234060   0.256560   0.277472
0.286270
     0.292916   0.302916   0.305224   0.305743   0.306376
0.307715
     0.315827   0.341357   0.353567   0.367206   0.386008
0.424947
     0.435969   0.498045   0.528106   0.733717   0.822942
0.855689

 Minimum Search - Taking Simple RFO Step
 Searching for Lamda that Minimizes Along All modes
 Value Taken    Lamda =  -0.00000048
 Step Taken.  Stepsize is  0.001284
```

```
                  Maximum     Tolerance    Cnvgd?
Gradient         0.000207      0.000300     YES
Displacement      0.000516      0.001200     YES
Energy change    -0.000004      0.000001      NO
```

```
#####################################################
#  Entering anlman.exe on Sun May 20 04:25:26 2012  #
#####################################################

 Analysis of SCF Wavefunction

 Total job wall time:  2.1e+006 s
```

12a D TAUTOMER

```
Spartan '02 Mechanics Program:  (PC/x86)
Release  115B

  Reason for exit: Successful completion
  Mechanics CPU Time : 000:00:00.2
  Mechanics Wall Time: 000:00:00.2
Spartan '02 Quantum Mechanics Program:  (PC/x86)
Release  115B
  To use a standard Psuedopotential a heavy atom must exists
  (An atom larger than 'Ar')

Job type: Geometry optimization.
Method: RB3LYP
Basis set: LACVP*
Number of shells: 90
Number of basis functions: 198

SCF model:
 A restricted hybrid HF-DFT SCF calculation will be
 performed using Pulay DIIS extrapolation


Optimization:
       Step     Energy        Max Grad.     Max Dist.
         1   -627.8047267     0.050776      0.142958
         2   -627.8199879     0.019256      0.057535
         3   -627.8219336     0.008723      0.022299
         4   -627.8222923     0.002632      0.012604
         5   -627.8223695     0.000885      0.003808
         6   -627.8223734     0.000400      0.001755
         7   -627.8223743     0.000099      0.000422
  Reason for exit: Sucessful completion
  Quantum Mechanics Program CPU Time : 000:37:01.5
  Quantum Mechanics Program Wall Time: 000:37:43.9
```

```
Spartan '02 Properties Program:  (PC/x86)
Release  115B
   Orientation rotated by    89.9999974955219     degrees!
  Reason for exit: Successful completion
  Properties Program CPU Time : 000:00:00.5
  Properties Program Wall Time: 000:00:00.6

** GEOMETRY OPTIMIZATION IN DELOCALIZED INTERNAL COORDINATES
**
   Searching for a Minimum

   Optimization Cycle:   7

                     Coordinates (Angstroms)
     ATOM              X           Y           Z
    1  N         1.993750   -0.073081    0.000000
    2  C         1.817724    1.372978    0.000000
    3  C         0.279588    1.579745    0.000000
    4  C        -0.276687    0.242032    0.000000
    5  C         0.804007   -0.732927    0.000000
    6  O         0.660693   -1.979211    0.000000
    7  O        -0.255063    2.675371    0.000000
    8  C        -1.581797   -0.225156    0.000000
    9  O        -1.842019   -1.513936    0.000000
   10  O        -2.607878    0.601768    0.000000
   11  C         3.283183   -0.727548    0.000000
   12  C        -3.934256    0.038606    0.000000
   13  H         2.264195    1.839033   -0.888406
   14  H         2.264195    1.839033    0.888406
   15  H        -0.923055   -1.983950    0.000000
   16  H         3.862968   -0.452893   -0.890611
   17  H         3.862968   -0.452893    0.890611
   18  H         3.114943   -1.805930    0.000000
   19  H        -4.601312    0.899820    0.000000
   20  H        -4.093074   -0.570432   -0.893084
   21  H        -4.093074   -0.570432    0.893084
   Point Group: cs   Number of degrees of freedom:  36


   Energy is   -627.822374316

 Hessian Updated using BFGS Update
  internal optimization with constraints (0)

 36 Hessian modes will be used to form the next step
  Hessian Eigenvalues:
     0.052360    0.056146    0.071671    0.075526    0.124610
0.129119
     0.131520    0.164593    0.170317    0.172341    0.192514
0.206383
```

```
    0.208965   0.217502   0.227339   0.241930   0.249711
0.260839
    0.285305   0.293983   0.302504   0.304684   0.306401
0.307676
    0.309849   0.312836   0.331040   0.355012   0.374657
0.411729
    0.479748   0.564567   0.628788   0.809205   0.859283
0.871055

 Minimum Search - Taking Simple RFO Step
 Searching for Lamda that Minimizes Along All modes
 Value Taken    Lamda =  -0.00000016
 Step Taken.  Stepsize is  0.000886

                      Maximum    Tolerance   Cnvgd?
        Gradient       0.000099     0.000300    YES
        Displacement    0.000422     0.001200    YES
        Energy change  -0.000001     0.000001    YES


#####################################################
#  Entering anlman.exe on Sun May 20 03:49:51 2012  #
#####################################################

 Analysis of SCF Wavefunction

 Total job wall time:  2.3e+006 s
```

12b A TAUTOMER

```
Spartan '02 Mechanics Program:  (PC/x86)
Release  115B

  Reason for exit: Successful completion
  Mechanics CPU Time : 000:00:00.1
  Mechanics Wall Time: 000:00:00.1
Spartan '02 Quantum Mechanics Program:  (PC/x86)
Release  115B
  To use a standard Psuedopotential a heavy atom must exists
  (An atom larger than 'Ar')

Job type: Geometry optimization.
Method: RB3LYP
Basis set: LACVP*
Number of shells: 102
Number of basis functions: 228

SCF model:
 A restricted hybrid HF-DFT SCF calculation will be
 performed using Pulay DIIS extrapolation
```

```
Optimization:
       Step     Energy        Max Grad.     Max Dist.
          1   -741.1674797      0.033171      0.128301
          2   -741.1764991      0.009202      0.028039
          3   -741.1773037      0.002734      0.009530
          4   -741.1773840      0.001031      0.003231
          5   -741.1773947      0.000449      0.001509
          6   -741.1773927      0.000083      0.002802
          7   -741.1773938      0.000042      0.002079
          8   -741.1773947      0.000044      0.000193
  Reason for exit: Sucessful completion
  Quantum Mechanics Program CPU Time : 000:41:30.4
  Quantum Mechanics Program Wall Time: 000:41:55.8

Spartan '02 Properties Program:  (PC/x86)
Release  115B
   Orientation rotated by   179.999994991044     degrees!
  Reason for exit: Successful completion
  Properties Program CPU Time : 000:00:00.6
  Properties Program Wall Time: 000:00:00.6

** GEOMETRY OPTIMIZATION IN DELOCALIZED INTERNAL COORDINATES
**
   Searching for a Minimum

   Optimization Cycle:   8

                  Coordinates (Angstroms)
    ATOM            X          Y          Z
     1  N        1.647789   0.259117  -0.000015
     2  C        1.365409   1.693397   0.000044
     3  C       -0.130732   1.717536  -0.000006
     4  C       -0.650996   0.453047  -0.000080
     5  C        0.455433  -0.523824  -0.000094
     6  O        0.413269  -1.737247  -0.000182
     7  O       -0.787264   2.857476   0.000028
     8  C       -2.093978   0.259808  -0.000122
     9  O       -2.868677   1.225690  -0.000332
    10  O       -2.505018  -1.005504   0.000127
    11  C        2.983058  -0.161660   0.000056
    12  C       -3.930756  -1.208452   0.000102
    13  C        3.262413  -1.645441  -0.000019
    14  O        3.861216   0.686217   0.000188
    15  H        1.785662   2.189170   0.882623
    16  H        1.785726   2.189251  -0.882458
    17  H       -1.754409   2.601206  -0.000039
    18  H       -4.379602  -0.762892  -0.891051
    19  H       -4.063394  -2.289821   0.000295
    20  H       -4.379676  -0.762563   0.891053
    21  H        2.819318  -2.129845   0.874496
```

```
  22  H         2.819385   -2.129744   -0.874624
  23  H         4.345825   -1.774922    0.000009
  Point Group: c1   Number of degrees of freedom:  63


  Energy is   -741.177394694

 Hessian Updated using BFGS Update
  internal optimization with constraints (0)

 63 Hessian modes will be used to form the next step
  Hessian Eigenvalues:
     0.001217   0.001855   0.004788   0.006954   0.008917
0.013630
     0.014707   0.016688   0.017941   0.021617   0.028454
0.037869
     0.044156   0.051300   0.054304   0.055285   0.055839
0.064094
     0.073472   0.074551   0.076415   0.096498   0.122129
0.128392
     0.147969   0.150825   0.166107   0.169468   0.176281
0.182830
     0.186001   0.199181   0.211768   0.215132   0.229340
0.241369
     0.243419   0.259385   0.273933   0.280443   0.285702
0.296180
     0.304598   0.305229   0.305467   0.305788   0.306029
0.306725
     0.307411   0.307420   0.311098   0.341966   0.349063
0.363231
     0.415959   0.457555   0.490336   0.532470   0.586210
0.675908
     0.801836   0.811903   0.856555

 Minimum Search - Taking Simple RFO Step
 Searching for Lamda that Minimizes Along All modes
 Value Taken    Lamda =  -0.00000003
 Step Taken.  Stepsize is  0.000598

                       Maximum    Tolerance   Cnvgd?
       Gradient         0.000044     0.000300    YES
       Displacement      0.000193     0.001200    YES
       Energy change    -0.000001     0.000001    YES


#####################################################
#  Entering anlman.exe on Tue May 22 07:55:47 2012  #
#####################################################

 Analysis of SCF Wavefunction
```

```
 Total job wall time:  2.5e+006 s
```

12b B TAUTOMER

```
Spartan '02 Mechanics Program:  (PC/x86)
Release  115B

  Reason for exit: Successful completion
  Mechanics CPU Time : 000:00:00.1
  Mechanics Wall Time: 000:00:00.1
Spartan '02 Quantum Mechanics Program:  (PC/x86)
Release  115B
  To use a standard Psuedopotential a heavy atom must exists
  (An atom larger than 'Ar')

Job type: Geometry optimization.
Method: RB3LYP
Basis set: LACVP*
Number of shells: 102
Number of basis functions: 228

SCF model:
 A restricted hybrid HF-DFT SCF calculation will be
 performed using Pulay DIIS extrapolation


Optimization:
       Step     Energy        Max Grad.     Max Dist.
          1   -741.1503465     0.052493      0.144245
          2   -741.1662310     0.013680      0.041460
          3   -741.1680629     0.007174      0.021750
          4   -741.1683874     0.002757      0.013853
          5   -741.1684544     0.001281      0.005085
          6   -741.1684670     0.000431      0.001458
          7   -741.1684682     0.000070      0.000201
  Reason for exit: Sucessful completion
  Quantum Mechanics Program CPU Time : 000:49:54.0
  Quantum Mechanics Program Wall Time: 000:50:40.5

Spartan '02 Properties Program:  (PC/x86)
Release  115B
   Orientation rotated by   89.9999974955219     degrees!
  Reason for exit: Successful completion
  Properties Program CPU Time : 000:00:00.7
  Properties Program Wall Time: 000:00:00.9


** GEOMETRY OPTIMIZATION IN DELOCALIZED INTERNAL COORDINATES
**
   Searching for a Minimum

   Optimization Cycle:   7
```

```
                    Coordinates (Angstroms)
     ATOM            X           Y           Z
    1  N         1.669146    0.295325    0.000000
    2  C         1.363475    1.729778    0.000000
    3  C        -0.151503    1.763041    0.000000
    4  C        -0.622677    0.419039    0.000000
    5  C         0.500538   -0.520573    0.000000
    6  O         0.490801   -1.737560    0.000000
    7  O        -0.855570    2.794275    0.000000
    8  C        -1.997862    0.187354    0.000000
    9  O        -2.834740    1.195671    0.000000
   10  O        -2.498179   -1.024590    0.000000
   11  C         3.011507   -0.101908    0.000000
   12  C        -3.935417   -1.174025    0.000000
   13  C         3.323458   -1.580150    0.000000
   14  O         3.875890    0.760588    0.000000
   15  H         1.777568    2.229458   -0.882404
   16  H         1.777568    2.229458    0.882404
   17  H        -2.256891    2.051555    0.000000
   18  H        -4.364798   -0.716233   -0.893787
   19  H        -4.100763   -2.250191    0.000000
   20  H        -4.364798   -0.716233    0.893787
   21  H         2.891774   -2.074780    0.874360
   22  H         2.891774   -2.074780   -0.874360
   23  H         4.409699   -1.684520    0.000000
   Point Group: cs    Number of degrees of freedom:  40


   Energy is   -741.168468236

 Hessian Updated using BFGS Update
  internal optimization with constraints (0)

 40 Hessian modes will be used to form the next step
  Hessian Eigenvalues:
     0.050755    0.056001    0.073878    0.075686    0.125084
0.128347
     0.129873    0.152817    0.161654    0.171025    0.172149
0.194800
     0.203963    0.214973    0.232089    0.234337    0.236113
0.251072
     0.257105    0.279808    0.280415    0.287861    0.303649
0.305207
     0.305650    0.307152    0.308287    0.317231    0.320475
0.364806
     0.392777    0.414562    0.457128    0.578729    0.610294
0.615740
     0.797565    0.811343    0.856214    0.873977

 Minimum Search - Taking Simple RFO Step
```

```
 Searching for Lamda that Minimizes Along All modes
 Value Taken    Lamda =  -0.00000008
 Step Taken.  Stepsize is  0.000557

                     Maximum     Tolerance    Cnvgd?
       Gradient        0.000070      0.000300     YES
       Displacement     0.000201      0.001200     YES
       Energy change   -0.000001      0.000001      NO


#####################################################
#  Entering anlman.exe on Sun May 20 09:19:56 2012  #
#####################################################

 Analysis of SCF Wavefunction

 Total job wall time:  3e+006 s
```

12b C TAUTOMER

```
Spartan '02 Mechanics Program:  (PC/x86)
Release  115B

  Reason for exit: Successful completion
  Mechanics CPU Time : 000:00:00.1
  Mechanics Wall Time: 000:00:00.2
Spartan '02 Quantum Mechanics Program:  (PC/x86)
Release  115B
  To use a standard Psuedopotential a heavy atom must exists
  (An atom larger than 'Ar')

Job type: Geometry optimization.
Method: RB3LYP
Basis set: LACVP*
Number of shells: 102
Number of basis functions: 228

SCF model:
 A restricted hybrid HF-DFT SCF calculation will be
 performed using Pulay DIIS extrapolation


Optimization:
       Step     Energy        Max Grad.     Max Dist.
          1   -741.1450738     0.036836      0.129532
          2   -741.1603094     0.013328      0.048885
          3   -741.1621251     0.003855      0.014685
          4   -741.1623188     0.002042      0.005652
          5   -741.1623338     0.001410      0.002419
          6   -741.1623422     0.000142      0.000820
  Reason for exit: Sucessful completion
```

```
  Quantum Mechanics Program CPU Time : 000:43:16.4
  Quantum Mechanics Program Wall Time: 000:43:47.5

Spartan '02 Properties Program:  (PC/x86)
Release  115B
   Orientation rotated by    89.9999974955219     degrees!
  Reason for exit: Successful completion
  Properties Program CPU Time : 000:00:00.6
  Properties Program Wall Time: 000:00:00.7


** GEOMETRY OPTIMIZATION IN DELOCALIZED INTERNAL COORDINATES
**
   Searching for a Minimum

   Optimization Cycle:   6

                    Coordinates (Angstroms)
     ATOM              X           Y           Z
    1  N         1.501701   -0.226595    0.000000
    2  C         1.323387    1.236774    0.000000
    3  C        -0.211749    1.449081    0.000000
    4  C        -0.782291    0.116785    0.000000
    5  C         0.256696   -0.818463    0.000000
    6  O         0.114626   -2.114239    0.000000
    7  O        -0.728105    2.551010    0.000000
    8  C        -2.148812   -0.357836    0.000000
    9  O        -2.425643   -1.569335    0.000000
   10  O        -3.081246    0.594115    0.000000
   11  C         2.747329   -0.896885    0.000000
   12  C        -4.446843    0.140969    0.000000
   13  C         3.958331    0.017623    0.000000
   14  O         2.830154   -2.105027    0.000000
   15  H         1.766380    1.693310   -0.890110
   16  H         1.766380    1.693310    0.890110
   17  H        -0.885213   -2.255821    0.000000
   18  H        -5.047430    1.050261    0.000000
   19  H        -4.652016   -0.457834   -0.890884
   20  H        -4.652016   -0.457834    0.890884
   21  H         3.974293    0.664745    0.884181
   22  H         4.847792   -0.612862    0.000000
   23  H         3.974293    0.664745   -0.884181
   Point Group: cs    Number of degrees of freedom:  40


   Energy is   -741.162342181

 Hessian Updated using BFGS Update
  internal optimization with constraints (0)

 40 Hessian modes will be used to form the next step
```

```
  Hessian Eigenvalues:
     0.051303   0.054956   0.068885   0.073387   0.122163
0.129109
     0.152342   0.170148   0.174695   0.180318   0.197616
0.208309
     0.215447   0.225991   0.230649   0.238333   0.244256
0.253523
     0.271320   0.279828   0.289447   0.295461   0.305096
0.305767
     0.306807   0.307358   0.310350   0.324279   0.337459
0.342455
     0.415601   0.460079   0.492472   0.504449   0.560668
0.602682
     0.786431   0.815408   0.831925   0.846594

 Minimum Search - Taking Simple RFO Step
 Searching for Lamda that Minimizes Along All modes
 Value Taken    Lamda =  -0.00000038
 Step Taken.  Stepsize is  0.001470

                        Maximum    Tolerance   Cnvgd?
        Gradient         0.000142     0.000300    YES
        Displacement      0.000820     0.001200    YES
        Energy change    -0.000008     0.000001     NO


#####################################################
#  Entering anlman.exe on Sun May 20 08:28:19 2012  #
#####################################################

 Analysis of SCF Wavefunction

 Total job wall time:  2.6e+006 s
```

12b D TAUTOMER

```
Spartan '02 Mechanics Program:  (PC/x86)
Release  115B

  Reason for exit: Successful completion
  Mechanics CPU Time : 000:00:00.3
  Mechanics Wall Time: 000:00:00.3
Spartan '02 Quantum Mechanics Program:  (PC/x86)
Release  115B
  To use a standard Psuedopotential a heavy atom must exists
  (An atom larger than 'Ar')

Job type: Geometry optimization.
Method: RB3LYP
Basis set: LACVP*
Number of shells: 102
```

```
Number of basis functions: 228

SCF model:
 A restricted hybrid HF-DFT SCF calculation will be
 performed using Pulay DIIS extrapolation


Optimization:
       Step     Energy        Max Grad.     Max Dist.
         1   -741.1491326      0.052301      0.143188
         2   -741.1652810      0.013337      0.041945
         3   -741.1671069      0.005876      0.016421
         4   -741.1673897      0.002968      0.009496
         5   -741.1674386      0.000850      0.002454
         6   -741.1674457      0.000249      0.000870
  Reason for exit: Sucessful completion
  Quantum Mechanics Program CPU Time : 000:46:03.0
  Quantum Mechanics Program Wall Time: 000:46:51.1

Spartan '02 Properties Program:  (PC/x86)
Release  115B
   Orientation rotated by   89.9999974955219     degrees!
  Reason for exit: Successful completion
  Properties Program CPU Time : 000:00:00.8
  Properties Program Wall Time: 000:00:01.0


** GEOMETRY OPTIMIZATION IN DELOCALIZED INTERNAL COORDINATES
**
   Searching for a Minimum

   Optimization Cycle:   6

                Coordinates (Angstroms)
     ATOM           X           Y           Z
    1  N        1.632642    0.472492    0.000000
    2  C        1.197822    1.878034    0.000000
    3  C       -0.341395    1.811961    0.000000
    4  C       -0.667754    0.395816    0.000000
    5  C        0.542373   -0.392665    0.000000
    6  O        0.599224   -1.639736    0.000000
    7  O       -1.066503    2.790348    0.000000
    8  C       -1.893073   -0.263313    0.000000
    9  O       -1.962561   -1.575807    0.000000
   10  O       -3.023284    0.404659    0.000000
   11  C        3.006546    0.169948    0.000000
   12  C       -4.261890   -0.336768    0.000000
   13  C        3.421830   -1.282813    0.000000
   14  O        3.803079    1.091484    0.000000
   15  H        1.572622    2.402840   -0.883821
   16  H        1.572622    2.402840    0.883821
```

```
  17  H        -0.996379  -1.910883   0.000000
  18  H        -4.332879  -0.960409   0.893903
  19  H        -5.039495   0.425511   0.000000
  20  H        -4.332879  -0.960409  -0.893903
  21  H         3.028413  -1.806417   0.875796
  22  H         3.028413  -1.806417  -0.875796
  23  H         4.512505  -1.310296   0.000000
  Point Group: cs   Number of degrees of freedom:  40


  Energy is   -741.167445698

 Hessian Updated using BFGS Update
  internal optimization with constraints (0)

 40 Hessian modes will be used to form the next step
  Hessian Eigenvalues:
     0.050649   0.055980   0.074991   0.075656   0.125061
0.128543
     0.147487   0.151569   0.169563   0.171516   0.176625
0.195465
     0.201046   0.213746   0.227332   0.233822   0.238801
0.252288
     0.261323   0.276251   0.282997   0.298527   0.304141
0.305203
     0.305970   0.306863   0.307947   0.312678   0.321083
0.365209
     0.396169   0.407953   0.465660   0.537983   0.590756
0.601848
     0.788391   0.814588   0.858671   0.874017

 Minimum Search - Taking Simple RFO Step
 Searching for Lamda that Minimizes Along All modes
 Value Taken    Lamda =  -0.00000087
 Step Taken.  Stepsize is  0.002106

                     Maximum    Tolerance   Cnvgd?
       Gradient       0.000249     0.000300    YES
       Displacement    0.000870     0.001200    YES
       Energy change   -0.000007     0.000001     NO


#####################################################
#  Entering anlman.exe on Sun May 20 07:43:28 2012  #
#####################################################

 Analysis of SCF Wavefunction

 Total job wall time:  2.8e+006 s
```

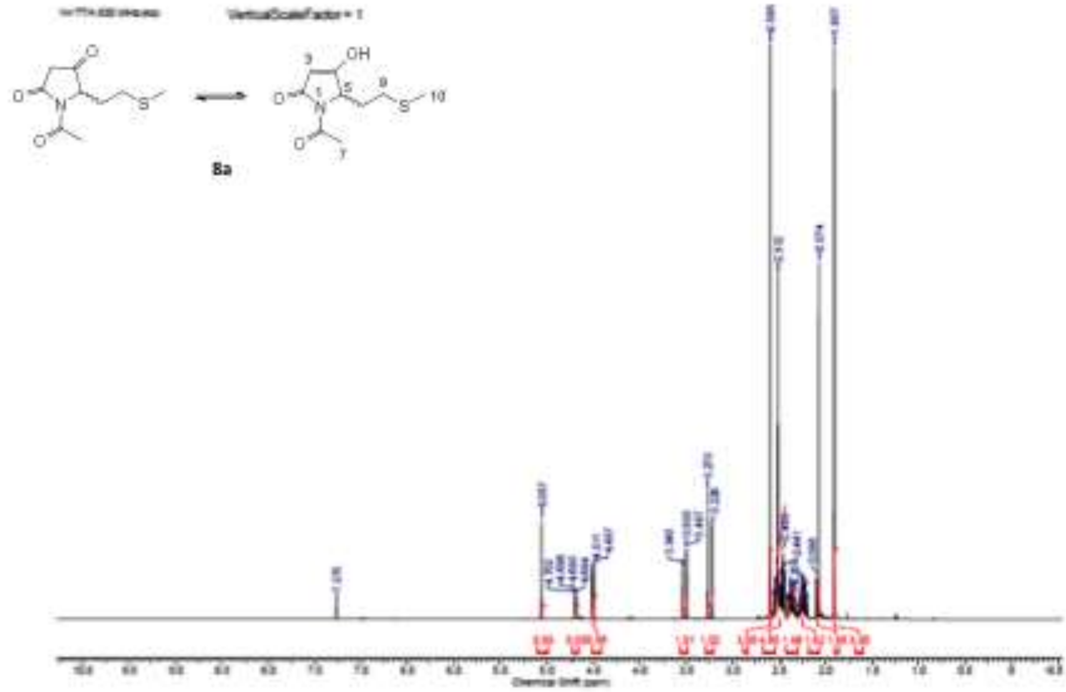
VerticalScaleFactor = 1
OH
8a
Chemical Shift (ppm)

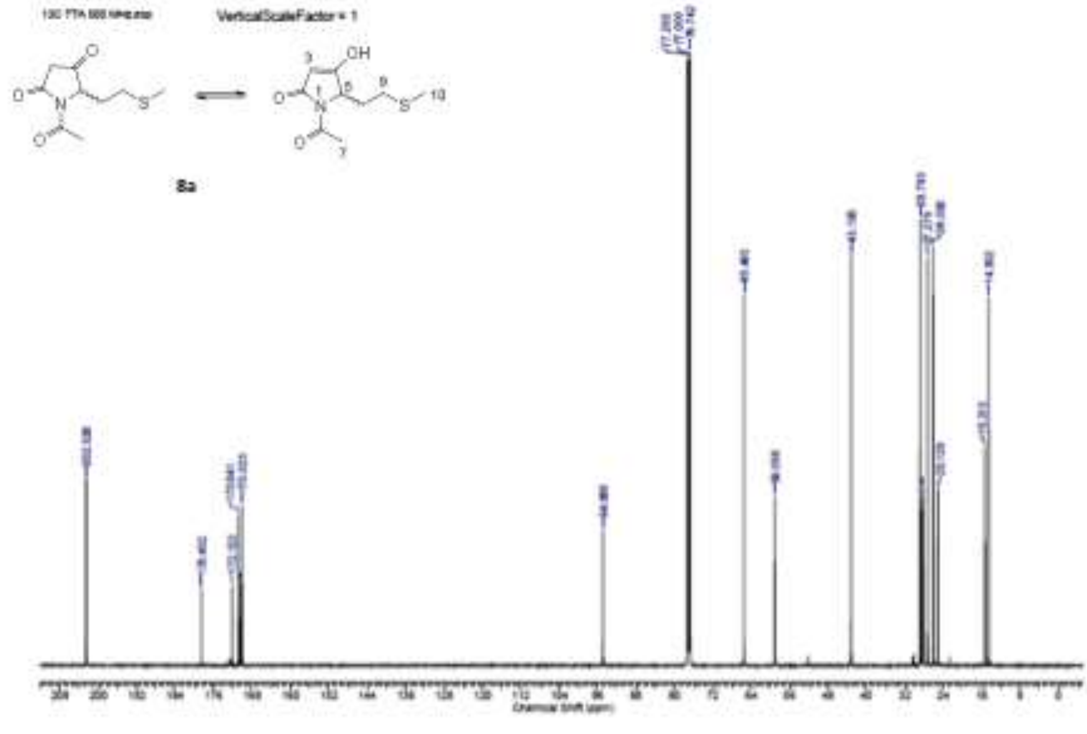
VerticalScaleFactor = 1
OH
8a
Chemical Shift (ppm)

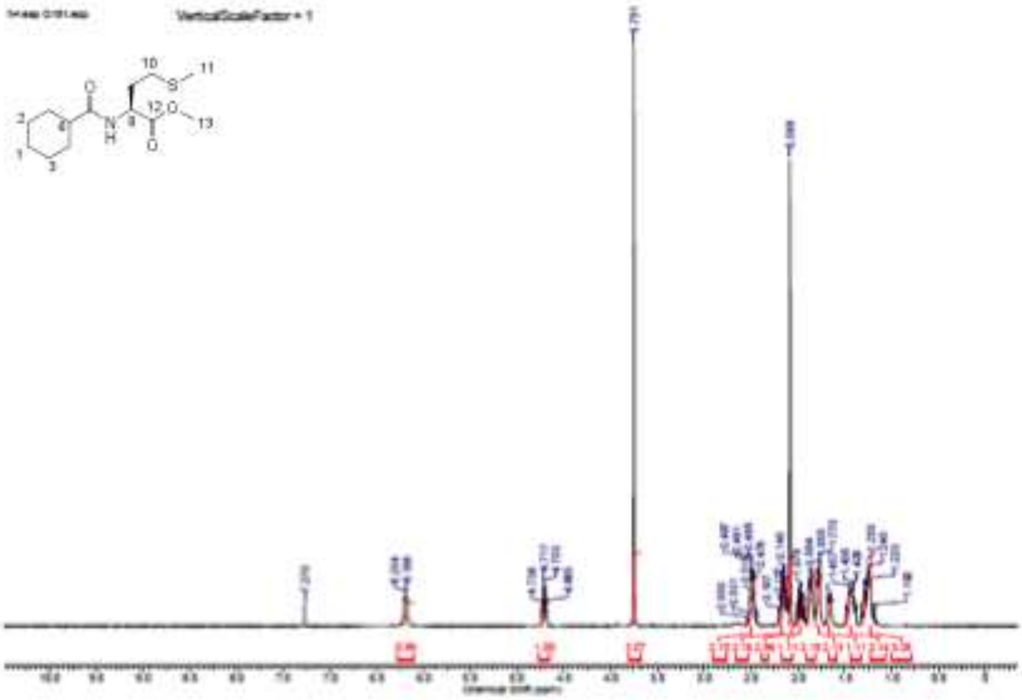

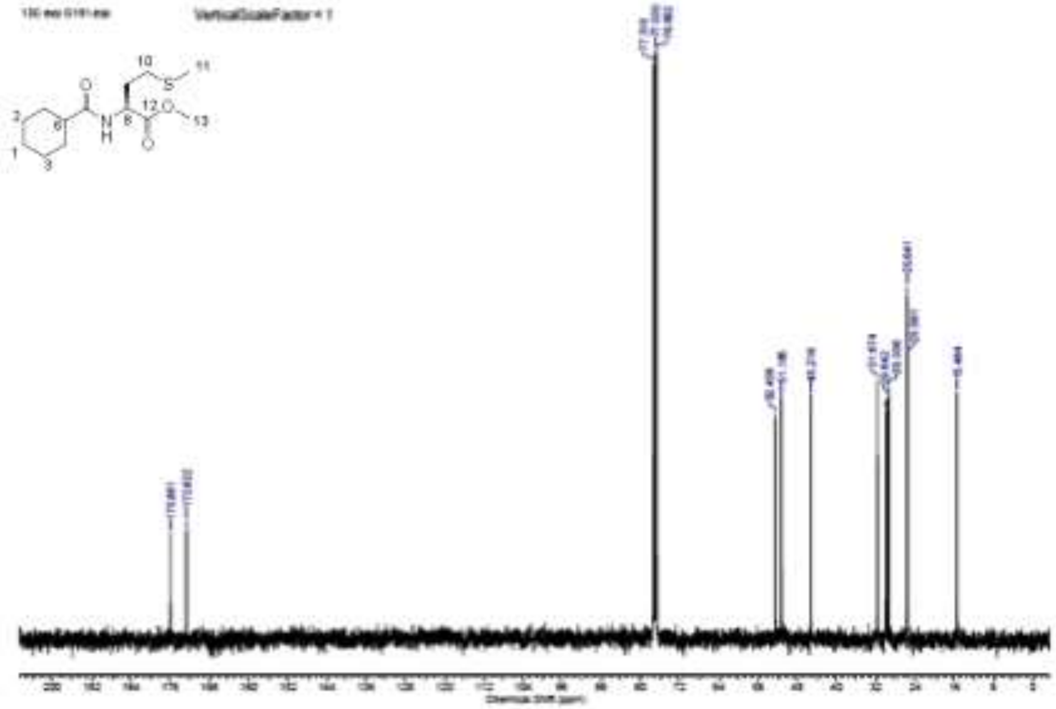

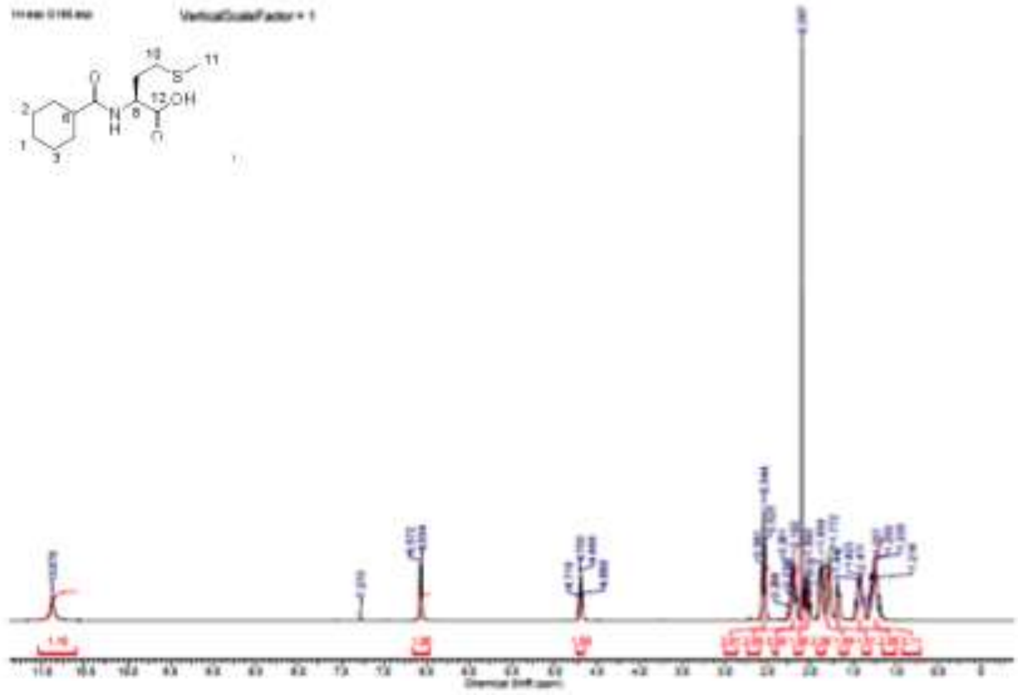

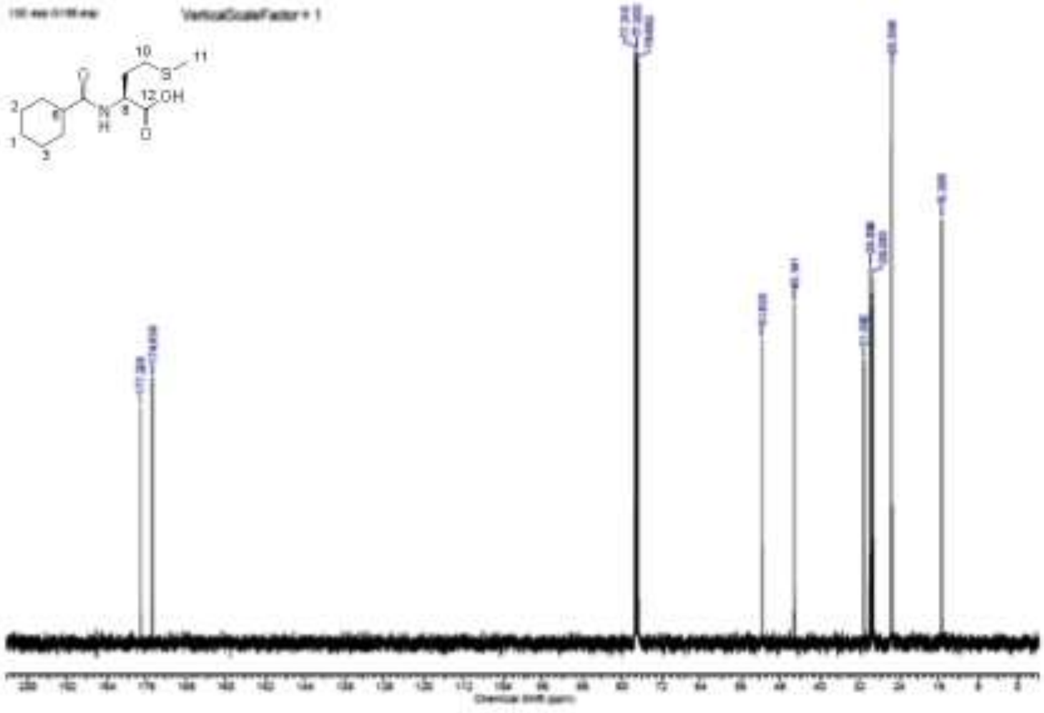

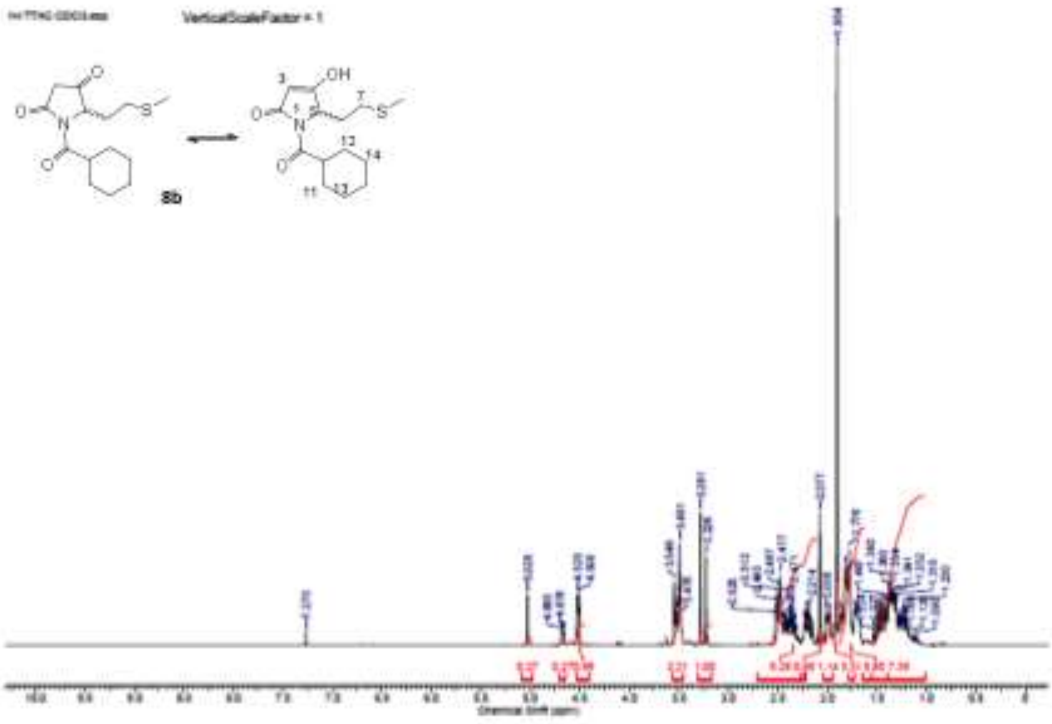

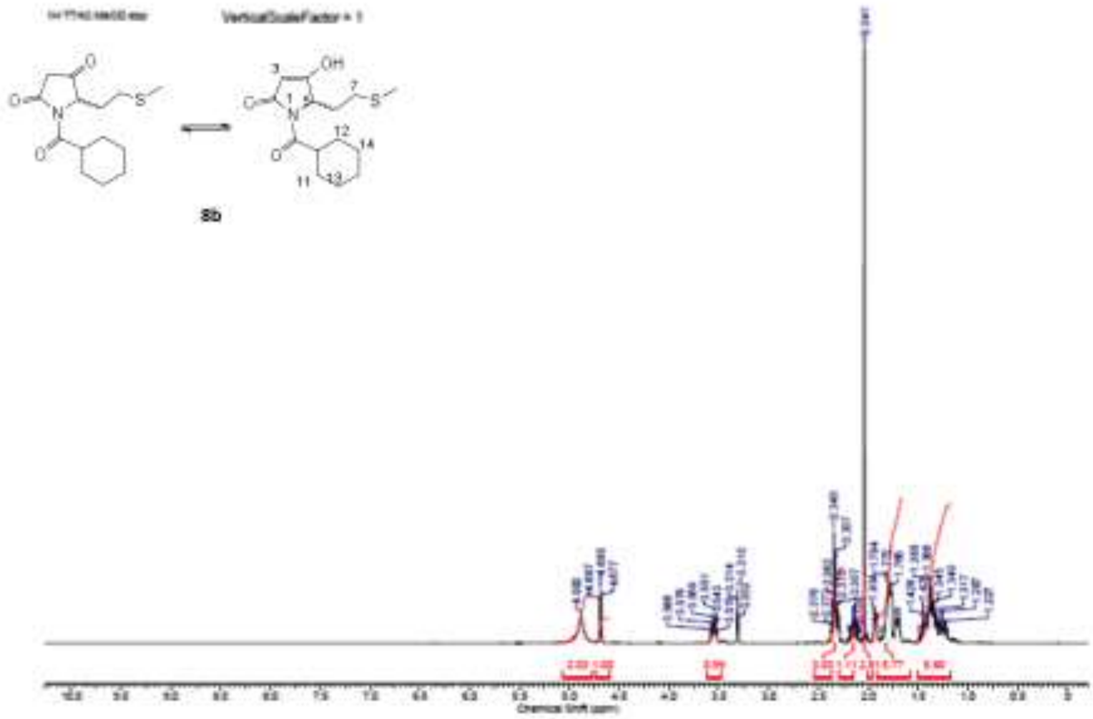

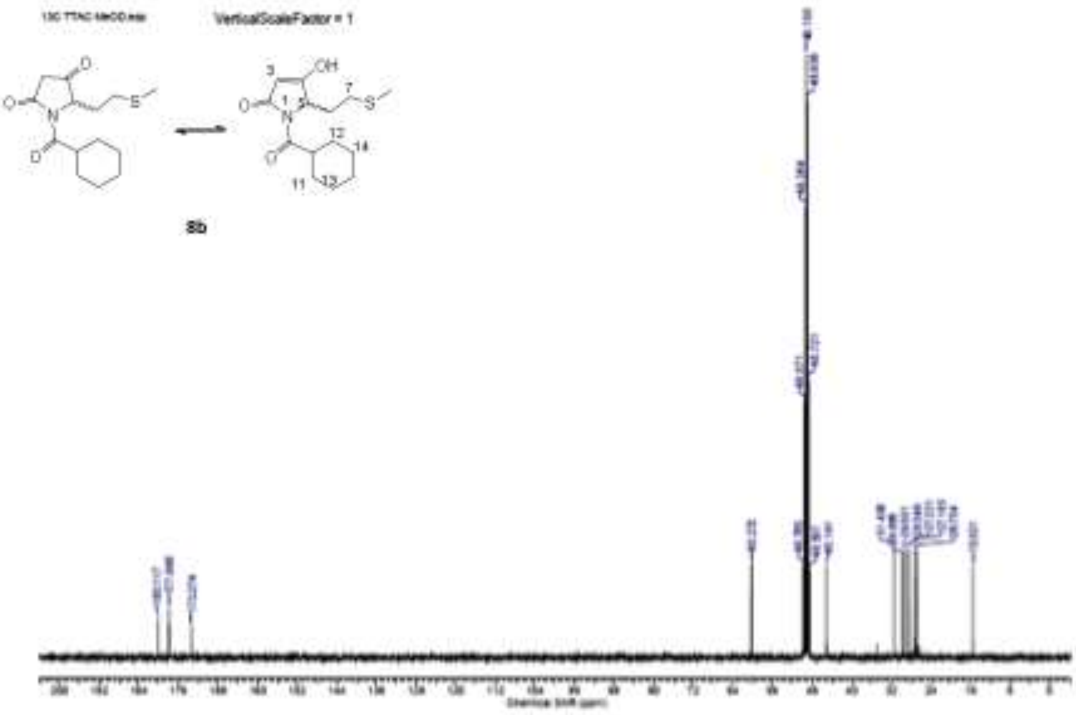

Table S1: LipE values of tetramates

	S1	S26	S4	S2	E1	E2	P1	P9	P9B	H3	H4
**2a**	3.21	2.91	2.91	2.91	2.91	2.91	2.61	2.91	2.31	2.61	3.21
**2b**	3.63	3.63	3.63	3.33	3.33	3.03	3.03	3.03	**-**	3.33	2.08
**2c**	3.31	3.31	3.31	3.31	3.31	3.31	3.00	3.31	3.31	3.60	4.81
**2d**	3.5	3.5	3.5	3.5	3.5	3.5	3.20	3.80	3.20	3.80	4.72
**2e**	2.37	1.46	1.46	1.46	1.76	2.91	2.07	2.67	2.07	0.86	3.67
**2f**	0.28	0.28	0.28	0.28	0.28	0.28	0.32	0.92	0.32	0.32	0.32
**2g**	3.13	3.13	3.13	3.13	2.83	3.13	2.83	2.22	2.83	3.13	3.43
**2h**	2.55	2.55	2.55	2.55	2.55	2.55	2.93	2.25	1.04	1.04	1.95
**3a**	2.92	2.92	3.23	2.92	3.53	3.53	3.83	3.83	3.83	2.92	2.92
**3b**	3.17	2.87	3.47	3.17	3.77	3.47	4.07	4.07	4.07	2.87	3.77
**3c**	3.82	3.22	3.22	3.53	3.82	3.53	3.82	3.82	3.82	3.22	4.13
**3d**	3.62	2.71	3.02	3.02	3.62	3.32	3.62	3.62	3.92	2.71	3.92
**3e**	2.97	2.97	2.97	2.97	2.97	2.97	2.97	2.97	2.97	2.97	2.97
**3f**	3.36	3.36	3.36	3.36	3.36	3.36	3.36	3.36	3.36	3.36	3.36

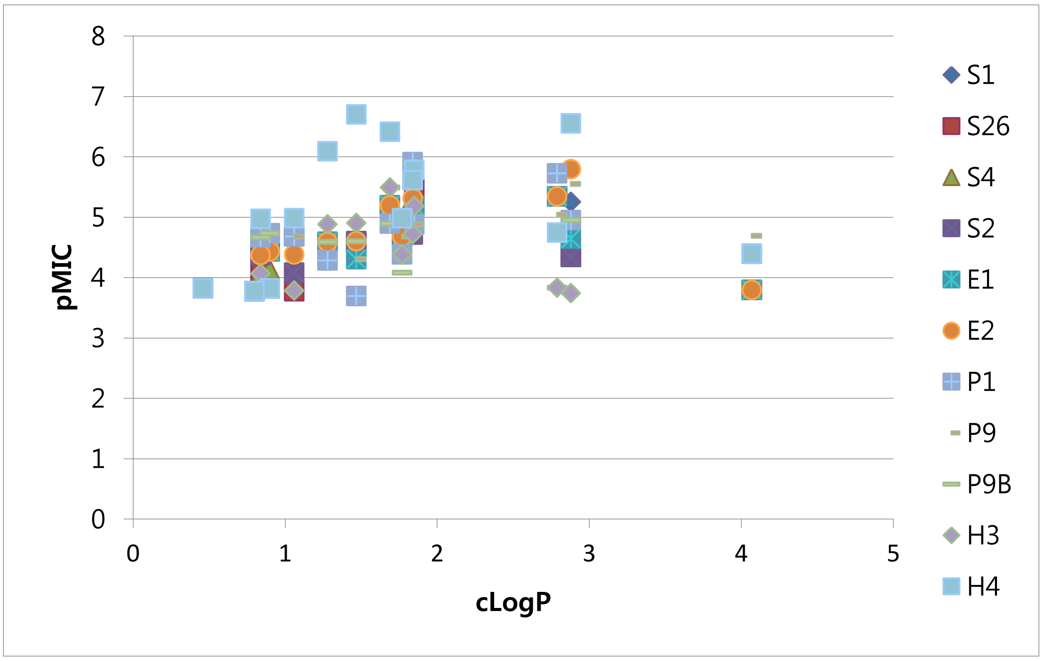

Figure S1: Plot of cLogP versus pMIC for the compounds shown in Table S1.
